# Progesterone Modulation of Pregnancy-Related Immune Responses

**DOI:** 10.3389/fimmu.2018.01293

**Published:** 2018-06-20

**Authors:** Nishel M. Shah, Nesrina Imami, Mark R. Johnson

**Affiliations:** ^1^Department of Surgery and Cancer, Imperial College London, Chelsea and Westminster Hospital, London, United Kingdom; ^2^Department of Medicine, Imperial College London, Chelsea and Westminster Hospital, London, United Kingdom

**Keywords:** pregnancy, progesterone, RU486, immune modulation, immune response

## Abstract

Progesterone (P4) is an important steroid hormone for the establishment and maintenance of pregnancy and its functional withdrawal in reproductive tissue is linked with the onset of parturition. However, the effects of P4 on adaptive immune responses are poorly understood. In this study, we took a novel approach by comparing the effects of P4 supplementation longitudinally, with treatment using a P4 antagonist mifepristone (RU486) in mid-trimester pregnancies. Thus, we were able to demonstrate the immune-modulatory functions of P4. We show that, in pregnancy, the immune system is increasingly activated (CD38, CCR6) with greater antigen-specific cytotoxic T cell responses (granzyme B). Simultaneously, pregnancy promotes a tolerant immune environment (IL-10 and regulatory-T cells) that gradually reverses prior to the onset of labor. P4 suppresses and RU486 enhances antigen-specific CD4 and CD8 T cell inflammatory cytokine (IFN-γ) and cytotoxic molecule release (granzyme B). P4 and RU486 effectively modulate immune cell-mediated interactions, by regulating differentiated memory T cell subset sensitivity to antigen stimulation. Our results indicate that P4 and RU486, as immune modulators, share a reciprocal relationship. These data unveil key contributions of P4 to the modulation of the maternal immune system and suggests targets for future modulation of maternal immune function during pregnancy.

## Introduction

Progesterone (P4) plays a key role in the establishment and maintenance of pregnancy and its withdrawal causes the onset of labor (1, 2). In humans, the production of P4 gradually rises throughout pregnancy; the corpus luteum is the major source in early pregnancy, but after 8 weeks, the placenta takes over (1). P4 and its metabolites work through a series of nuclear P4 (PR) and glucocorticoid (GR) receptors, as well as membrane-bound P4 receptors (mPR), to exert their effects (3–5). The majority of P4 actions on immune function are mediated by its interactions with GR (6–9). Human PBMCs are thought to express a number of endocrine receptors including: mPR, GR, and estrogen receptors (4, 6, 7, 10). In murine models, GR engagement increases regulatory T cell (Treg) immune-suppressive function, and P4 binding to GR is thought to be responsible for this finding in pregnancy (8, 9). In humans, GR agonists have been shown to increase apoptosis of CD4^+^ T cells *via* the GR (7). In contrast to most animal models of pregnancy, where there is a systemic withdrawal of P4 prior to the onset of labor, in the human, the literature suggests that there is a functional withdrawal at the level of the PR at the end of pregnancy (11). In particular, there are differences seen in the level of expression of P4 receptor isoforms, P4 receptor gene polymorphisms in reproductive tissue, and a decline in secretion of a lymphocyte-derived immunomodulatory protein known as P4-induced blocking factor (PIBF) (1, 11). P4 acts, either directly, or indirectly through PIBF, to modulate the immune system to achieve a successful pregnancy. These include promoting a T_H_2-dominant cytokine profile and upregulating HLA-G expression on trophoblast, which enables γδ T-cell activation and evasion of host defenses by acting as a ligand for inhibitory receptors on natural killer (NK) cells (1, 12–14). In fact, PIBF is a potent suppressor of cytotoxic immune cells and regulator of cytokine secretion (11, 15).

Pregnancy is associated with a series of immune adaptations that begin pre-implantation and span the length of the antenatal and postnatal period (16–18). As a result, maternal immune responses are different compared to non-pregnant women, and they fluctuate during the course of pregnancy (19, 20). In fact, some autoimmune conditions, such as rheumatoid arthritis, enter remission during pregnancy, but flare up in the postnatal period (21). Although clinical trials using HRT in women with rheumatoid arthritis have not shown the same effect as pregnancy, animal models using pregnancy-like levels of hormones have shown promising findings (22). In clinical practice, P4 supplementation is used in pregnancy as an effective treatment for the prevention of preterm birth (23). Its effects are likely to be a combination of immune modulation and a reversal of the functional withdrawal of P4 action in reproductive tissue. Spontaneous labor is associated with a loss of suppression of syngeneic and allogeneic T-cell responses (24). Interestingly, gestational changes are seen *ex vivo* where fetal-specific T effector memory (T_EM_) cells and detectable fetal DNA are longitudinally increased in pregnancy (18). Peripheral blood IFN-γ, IL-4 spontaneous responses, and paternal antigen stimulated responses, measured using enzyme-linked immunospot (ELISpot), appear to peak at 35 weeks of pregnancy (19).

We investigated the hypothesis that in the peripheral circulation during pregnancy, P4 actively suppresses CD4 and CD8 T cell inflammatory cytokine and cytotoxic molecule production. In addition, we hypothesized that P4 alters T cell, NK cell, and dendritic cell (DC) phenotype to regulate immune responses. To determine the effects of P4, we began by determining the gestational changes in immune responses and leukocyte phenotype, longitudinally, in healthy uncomplicated pregnancies, which we expected may be affected by the use of P4. We then compared this cohort of patients to those supplemented with vaginal P4. Finally, to understand the potential effects of P4 antagonism in a clinical setting, we recruited patients receiving the most widely used P4 antagonist mifepristone (RU486) in second trimester pregnancies and analyzed its effects longitudinally. Our study takes a novel approach by comparing the effects of P4 supplementation and the use of RU486 in pregnancy. Importantly, this is the first study to investigate the immune-modulatory effects of RU486, *in vivo*, in the mid second trimester of pregnancy. We found that advancing pregnancy may be associated with an inherent loss of sensitivity to P4. More importantly, P4 reduces pro-inflammatory and cytotoxic T cell responses. It achieves this by a combination of effects to modulate immune cell-mediated interactions, including memory T cell antigen sensitivity and regulation of leukocyte migration.

## Materials and Methods

### Ethics Statement

All subjects were recruited from Chelsea and Westminster Hospital, London, UK. This study was carried out in accordance with the recommendations of National Institute of Health Research (NIHR) Good Clinical Practice guidelines, and a NHS Research Ethics Committee. The protocol was approved by the National Research Ethics Service (NRES), London, UK committee as well as by Chelsea and Westminster NHS Trust, London, UK; Ref: 11/LO/0971. All subjects gave written informed consent in accordance with the Declaration of Helsinki.

### Study Design

Based on the premise that maternal peripheral blood will provide a window to the systemic effects of P4 in pregnancy, a combination of ELISpot, 9-parameter flow cytometry, and multiplex assays were developed to assess functional cellular responses and leukocyte phenotype. A standardized protocol was used for all samples to ensure consistency and comparability. Blood samples were processed within 2 h of collection and all phenotypic and functional work was performed on fresh samples.

### Study Participants

For the control group, healthy pregnant patients (*N* = 42) were recruited from the antenatal clinic from April 2013 to September 2014 at Chelsea and Westminster Hospital during their first visit before 20 weeks of gestation. These patients had no previous history of premature delivery defined as less than 37 weeks of gestation. Women with a history of previous vaginal deliveries were not excluded so that the sample population was comparable to the P4 supplemented cohort, which were often started on P4 due to a previous history. Once recruited, these patients were asked to provide peripheral blood samples longitudinally. The time points for sample collection were: at recruitment (range 11–20 weeks of gestation), 28, 34 weeks, in labor or at delivery, 24 h postnatal, and 6–8 weeks postnatal.

Pregnant patients receiving P4 supplementation (*N* = 15) (once a day Cyclogest^®^ 400 mg per vaginum/rectum, Actavis UK) were recruited from the preterm antenatal clinic at Chelsea and Westminster Hospital between April 2013 and September 2014, ideally prior to 20 weeks of gestation (range 17–20 weeks of gestation). These patients were commenced on P4 due to: a previous history of preterm labor and delivery, second trimester loss, or ultrasonographic evidence of cervical shortening of <25 mm, which carries a significantly increased risk of preterm delivery at ≤32 weeks of gestation (23). These patients continued P4 treatment until 34 weeks of gestation. For these patients, the time points for longitudinal sample collection were: at recruitment, 28, 34, and 36 weeks of gestation. Since exogenous vaginal P4 (100 mg tablets) has a terminal half-life of approximately 14 h, the effect of P4 was thought to have substantially declined by the 36 weeks of gestation (25). The effects of supplementary P4 should disappear in the postnatal period. Since treatment stopped at 34 weeks of gestation, no differences were expected between the P4 treated and control subjects, and so these were not compared.

In order to understand the effects of P4 antagonism, pregnant patients (*N* = 8) undergoing medically indicated terminations of pregnancy due to fetal anomaly in an otherwise uncomplicated pregnancy were recruited from the fetal medicine unit at Chelsea and Westminster Hospital, from June 2013 to May 2015. As part of the termination process, these patients received RU486 (once only Mifegyne^®^ 400 mg orally, Nordic Pharma UK) and then attended the hospital for prostaglandin-induced labor 2 days later. Once recruited, these patients were asked to provide peripheral blood samples prior to taking RU486, 48 and 72 h post RU486, which was 24 h post-delivery. The mean gestation at delivery was 19 weeks (SD ± 1.8).

Study inclusion criteria included: age at booking under 40 years as replicative senescence, immune exhaustion, and thymic output is age linked with the latter reduced beyond 40 years of age (26). Exclusion criteria included: failure to meet the inclusion criteria; the development of or past medical history of any pregnancy related or unrelated complications that affected the course of pregnancy, such as pre-eclampsia, gestational diabetes, or intra-uterine growth restriction as well as any autoimmune, hypertensive, or renal conditions. Demographic data are summarized in Table S1 in Supplementary Material.

Non-pregnant female control samples were obtained from individuals working in the Centre for Immunology and Vaccinology, Imperial College London, UK and clinical staff working in the Maternity Department, Chelsea and Westminster Hospital.

### Preparation of Cells

Approximately 35 ml of peripheral blood was obtained using a Vacutainer™ system and using 6 ml lithium heparin blood collection tubes (Becton Dickinson, Oxford, UK). PBMCs from adult peripheral blood were prepared by density gradient centrifuge on Histopaque (Sigma-Aldrich, Dorset, UK) as described previously (27). Viability was determined using a trypan blue exclusion test of cell viability and samples where this was greater than 80% were used. For functional work, the cells were suspended in TCM [RPMI-1640 with Penicillin and Streptomycin (Sigma-Aldrich), at final concentrations of 100 IU/ml and 100 µg/ml, and l-glutamine (Sigma-Aldrich) at a final concentration of 2 mM], and for phenotype the cells were suspended in Ca^2+^ and Mg^2+^ containing PBS (Sigma-Aldrich). All ELISpot and flow cytometry assays were performed on fresh samples. Processing was commenced within 2 h of obtaining peripheral blood. Plasma for multiplex immunoassay was collected, aliquoted, and stored at −80°C until use.

### ELISpot Assay

IFN-γ, IL-10, IL-4, and Granzyme B ELISpot assays were performed in order to detect recall antigen/peptide specific T cell responses as previously described (28). Briefly, 1 × 10^5^ PBMC/well were cultured in 10% (heat-inactivated) male AB plasma-RPMI (200 μl/well, Sigma-Aldrich) in 96-well polyvinylidene difluoride backed plates (Merck Millipore, Hertfordshire, UK), which were coated in antibodies for the cytokines and proteases of interest, namely IFN-γ, IL-10, Granzyme B, and IL-4 (MabTech, Nacka Strand, Sweden). PBMCs, in duplicate wells, were stimulated with 100 µl of an antigen/peptide pool obtained from NIBSC (NIBSC, Hertfordshire, UK) and Virion-Serion (Virion-Serion, Würzburg, Germany) at the manufacturer’s recommended concentrations. These included: EBV, CMV, influenza A, measles, and HSV whole lysates; purified protein derivative (PPD) of *M. Tuberculosis* Tuberculin; purified tetanus toxoid (TTOX); and flu/EBV/influenza (FEC) peptide pool. Positive and negative controls were provided by phytohemagglutinin (5 µg/ml) and TCM. The plate was incubated at 37°C in 5% CO_2_ for 48 h for anti IFN-γ, IL-10, and Granzyme B coated and 96 h for anti-IL-4 coated plates. Detection of spot forming cells (SFC) was carried out by the addition of biotinylated anti IFN-γ, IL-10, Granzyme B, or IL-4 (MabTech) and incubation, followed by the use of a concentrated streptavidin-alkaline phosphatase conjugate (MabTech). Finally, the development step required the use of a chromogen prepared from a premixed BCIP/NBT substrate kit (BioRad labouratories Ltd., Hertfordshire, UK). Spot reading and counting was performed using an AID ELISpot reader (Oxford Biosystems Cadama, Oxfordshire, UK).

### Flow Cytometric Leukocyte Quantification

Multicolor color flow cytometry was used to phenotype CD4 and CD8 T-cell subsets, NK cells, and DC. In order to evaluate T cell subtypes, PBMCs were stained with the following murine, anti-human monoclonal antibodies according to the manufacturer’s instructions: peridinin chlorophyll protein (PerCP) Cy5.5-labeled anti-CD3 (Biolegend, London, UK); allophyocyanin (APC)-H7-conjugated anti-CD8 (Biolegend); BD Horizon V450-labeled anti-CD38 (BD Biosciences, Oxford, UK), anti-CD127 (BD), and anti-CCR4 (BD); BD Horizon V500-labeled anti-HLA-DR (BD) and anti-CD4 (BD); fluorescein isothiocyanate (FITC)-labeled anti-CD31 (BD), anti-CD25 (BD), anti-CCR6 (R&D Systems, Abingdon, UK) and anti-PIBF (rabbit polyclonal; Biorbyt, Cambridge, UK); phycoerythrin (PE)-conjugated anti-CCR7 (R&D), anti-CCR5 (BD) and anti-CCR3 (BD); APC-labeled anti-CD28 (BD), anti-HLA-G (eBioscience, Cheshire, UK), anti-CXCR3 (BD); PECy7-labeled anti-CD45RA (BD), anti-CD45RO (BD) and anti-CXCR4 (BD). NK subsets were phenotyped with: BD Horizon V450-labeled anti-CD56 (BD); FITC-labeled anti-PIBF; PE-labeled anti-iNKT (BD); APC-labeled anti-TCR-γδ (BD); PE-Cy7-labeled anti-CD16 (BD); and PerCP-Cy 5.5-labeled anti-CD3. Approximately 2 × 10^6^ cells were stained per tube, incubated in the dark at room temperature for 30 min, washed with PBS, and fixed with BD stabilizing fixative (BD Biosciences), before acquisition within 24 h. At least 100,000 events were acquired on a 3-laser flow cytometer (BD Biosciences LSR II) and subsequently gated according to respective isotype controls.

Dendritic cell of myeloid and plasmacytoid lineage, as well as HLA-G expressing tolerant variants were identified using the antibodies: Qdot^®^ 605-labeled anti-CD3 and anti-CD19 (Invitrogen, Paisley, UK); BD Horizon V450-labeled anti-CD11c (BD); BD Horizon V500-labeled HLA-DR; FITC-labeled anti-CD16 (BD); PE-labeled anti-ILT4 (eBioscience); APC-labeled anti-HLA-G (eBioscience); PE-Cy7-labeled anti-CD83 (BD); PerCP-Cy 5.5-labeled CD123 (BD); and APC-H7-labeled anti-CD14 (BD). A minimum of 500,000 events were acquired according to the description detailed above. Analysis of flow cytometric data was performed using FlowJo version 7.65 (Tree Star Inc., Ashland, OR, USA).

### Multiplex Immunoassay

A human 17-plex Bio-Plex Pro^®^ Multiplex immunoassay kit (BioRad) was used to determine plasma cytokine and chemokine concentrations. The kit contained the following cytokines: G-CSF, GM-CSF, IFN-γ, IL-1β, IL-2, IL-4, IL-5, IL-6, IL-7, IL-8, IL-10, IL-12 (p70), IL-13, IL-17, MCP-1 (MCAF), MIP-1β, TNF-α. The multiplex immunoassay was performed according to the manufacturer’s instructions and assays were read using a Bio-Plex© MAGPIX© reader (BioRad).

### Statistics

Longitudinal analysis of un-supplemented pregnancies was undertaken using mixed-effects modeling to avoid a loss of statistical power by omitting patients with incomplete data. For normally distributed data, a linear mixed effects model was used, and pairwise multiple comparisons of estimated marginal means with sequential Bonferroni correction was performed where the main effect was significant. Where data did not follow a normal distribution, a generalized linear mixed effects model with gamma log-link was used. If the main effect was significant, pairwise multiple comparisons of estimated marginal means with sequential Bonferroni correction was performed. Data were analyzed following the methods outlined by Duricki et al. and using IBM© SPSS Version 21.0 (Armonk, New York, NY, USA) for mixed effects modeling (29). In addition, longitudinal ELISpot data presented as a heatmap was produced and the analysis and presentation of distributions was performed using SPICE version 5.1, downloaded from http://exon.niaid.nih.gov. Comparison of distributions was performed using a Student’s *t*-test and a partial permutation test as described (30).

P4 supplemented and un-supplemented pregnancies were compared using a Students *t*-test where the data were continuous and parametric, and for non-parametric data, Mann–Whitney *U* test was used. For longitudinal analysis of P4 and RU486 treated pregnancies with repeated measures and parametric data, a one-way analysis of variance (ANOVA) with multiple group comparisons and a Tukey correction was used to compare group means. For non-parametric longitudinal repeated measures, data analysis was undertaken using a Friedman test with Dunn’s correction. Statistical analysis was performed on GraphPad Prism version 6.0 (GraphPad Software, San Diego, CA, USA).

Data are presented as means ± SEM or medians ± interquartile range (IQR) as appropriate for the distribution normality. All *P*-values were two-tailed and significance was defined as *P* < 0.05.

## Results

### Pregnancy Promotes a Tolerant Immune Environment in the Peripheral Circulation That Gradually Reverses Prior to the Onset of Delivery

Before investigating the effects of P4, leukocyte functional and phenotypic profiles were analyzed longitudinally in un-supplemented pregnancies. This established the effects of pregnancy and advancing gestation as well as providing a control group. Baseline results from pregnant participants were compared with non-pregnant volunteers. Where longitudinal analysis in pregnancy was significant, pairwise analysis comparing with baseline, 34 weeks and labor were reported. These co-responded to recruitment, prior to the onset of labor and labor/delivery.

**Figure 1:**
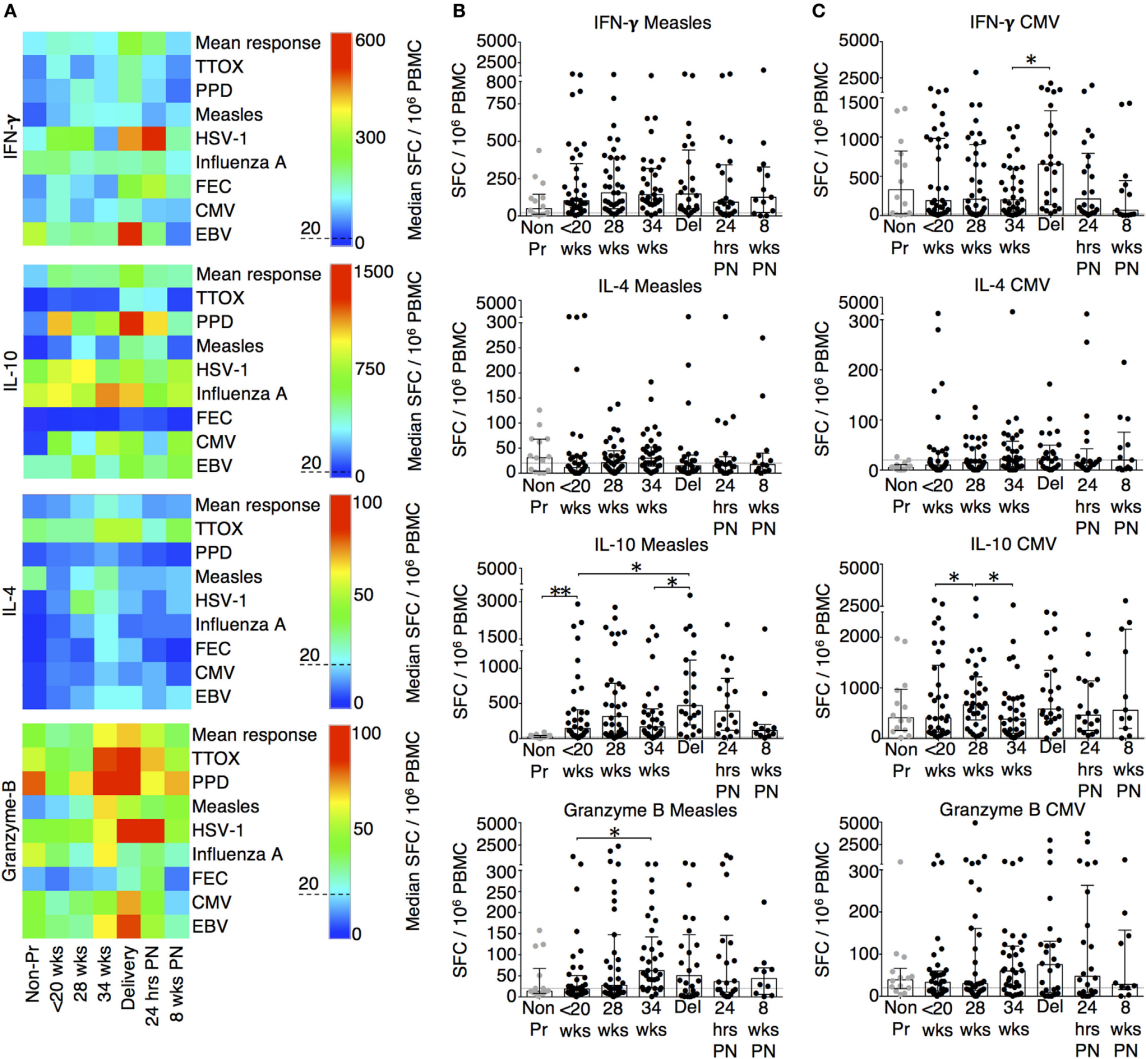
Advancing pregnancy is associated with greater IFN-γ, IL-10, IL-4, and granzyme B ELISpot responses. **(A)** Summary heat-map of gestation specific median ELISpot responses to antigens/peptides as well as overall mean response for each analyte. The individual scales used for each cytokine is shown with the threshold of 20 spot forming cells (SFC)/10^6^ PBMC indicated using a dashed line. The heat-map was generated and analyzed using SPICE (30). **(B)** ELISpot responses to measles and **(C)** CMV whole lysates. Columns indicate median and interquartile range. Gestation at sampling is indicated in pregnancy (●): < 20^+0^ weeks (*N* = *42*), 28 weeks (*N* = *35*), 34 weeks (*N* = *33*), at delivery (*N* = *24*), 24 h post-delivery (*N* = *22*), 6–8 weeks postnatal (*N* = *13*). Non-pregnant controls are depicted as 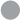 (*N* = *14*). A dashed line represents <20 SFC/10^6^. *P* values are two tailed and significance is defined as *P < 0.05 and **P < 0.01. Non-pregnant and baseline pregnant data analyzed by Mann–Whitney *U* test. Longitudinal data analyzed by generalized linear mixed effects model with gamma log-link and pairwise multiple comparisons of estimated marginal means with sequential Bonferroni correction.

T cell antigen-specific responses were determined using ELISpot and the results are summarized using the heat-map in Figure 1A. The majority of pregnant patients at baseline produced robust IFN-γ responses that were comparable to non-pregnant volunteers (Figures 1A–C; Figure S1 in Supplementary Material). Irrespective of the baseline response, labor and delivery was associated with a significant peak in cellular responses to CMV (Figure 1C) and HSV (Figure 1A). IL-10 cytokine responses behaved in a similar manner to IFN-γ. Our results showed that labor and delivery was characterized by a peak in responses to measles and CMV (Figures 1B,C) whole lysates, as well as TTOX (Figure S1 in Supplementary Material), PPD antigens, and FEC peptide pool (Figure 1A). Both IFN-γ and IL-10 responses returned to non-pregnant levels in the postnatal period. In contrast to IFN-γ, pregnancy was associated with improved IL-10 cellular responses throughout its course. The majority of IL-4 responses were below the positive threshold. When measurable, IL-4 response patterns were associated with an improved response to influenza A, PPD, TTOX, FEC peptide pool, and herpesvirus antigen (Figure 1A; Figure S1 in Supplementary Material). However, the peak response in pregnancy was at 34 weeks of gestation and the majority of these returned to non-pregnant levels in labor or 24 h post-delivery (Figure 1A). Most pregnant patients had positive cytotoxic granzyme B responses at baseline, but this was not true for all non-pregnant patients where some responses fell below the threshold of 20 per 10^6^ SFC. When compared to baseline, 34 weeks of pregnancy showed a significant peak in SFC and a positive response to measles whole lysate and TTOX antigen (Figure 1B; Figure S1 in Supplementary Material).

**Figure 2:**
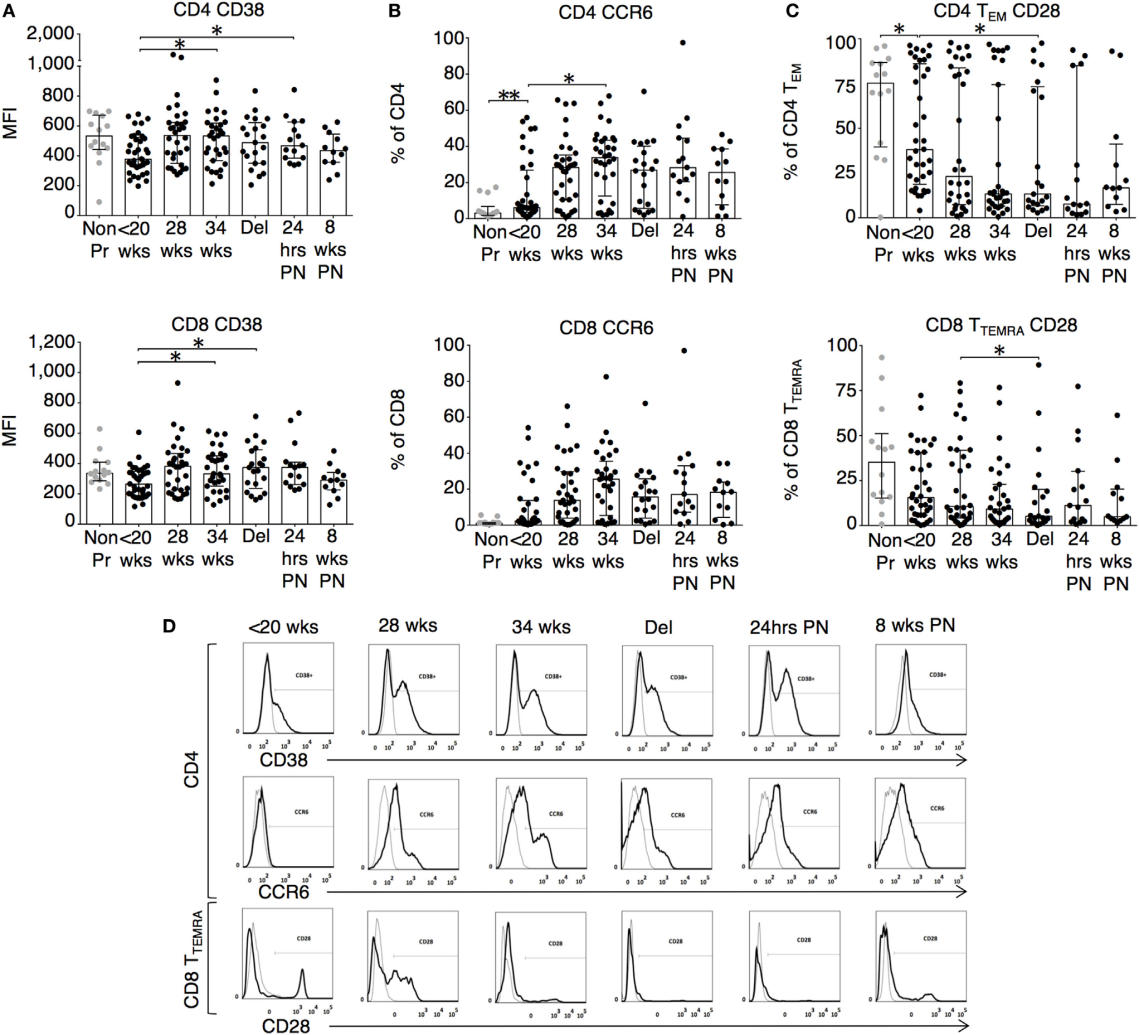
Leukocyte phenotype during pregnancy shows increased activation and migratory potential. **(A)** CD38 mean fluorescence intensity and the proportion of expression of **(B)** CCR6 on CD4 and CD8 T cells; **(C)** CD28 expression on CD4 T_EM_ (CCR7^−^CD45RA^−^) and CD8 T_TEMRA_ (CCR7^−^CD45RA^+^) T cell subtypes analyzed longitudinally with gestation and compared to controls. Gestation at sampling is indicated in pregnancy (●): <20^+0^ weeks (*N* = *42*), 28 weeks (*N* = *35*), 34 weeks (*N* = *33*), at delivery (*N* = *24*), 24 h post-delivery (*N* = *22*), 6–8 weeks postnatal (*N* = *13*). Non-pregnant controls are depicted as 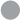 (*N* = *14*). *P* values are two tailed and significance is defined as *P < 0.05 and **P < 0.01. Non-pregnant and baseline pregnant data analyzed by Mann–Whitney *U* test. Longitudinal data analyzed by generalized linear mixed effects model with gamma log-link and pairwise multiple comparisons of estimated marginal means with sequential Bonferroni correction. **(D)** Shows representative flow cytometry histograms across gestations with matched isotype controls for CD38 and CCR6 expression on CD4 T cells as well as CD28 expression on CD8 T_TEMRA_ and HLA-DR expression on CD8 T cells.

**Figure 3:**
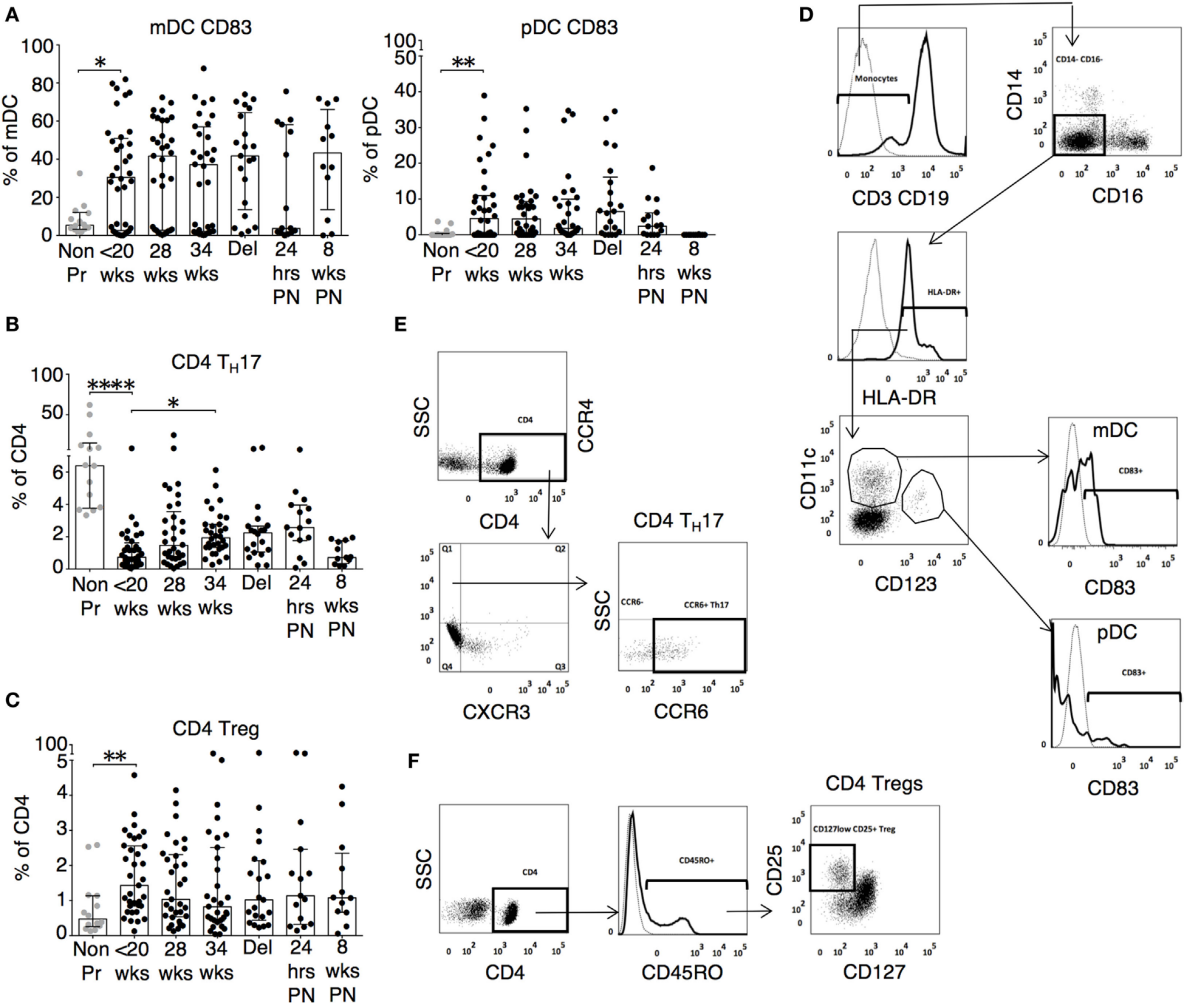
Inflammatory, activated, and cytotoxic leukocyte subsets show gestational variation in pregnancy. **(A)** Proportions of activated mDC (CD3^−^CD19^−^CD14^−^CD16^−^HLA-DR^+^CD11c^+^CD123^−^CD83^+^) and pDC (CD3^−^CD19^−^CD14^−^CD16^−^HLA-DR^+^CD11c^+^CD123^+^CD83^+^) during pregnancy and compared with gestation matched controls. **(B)** Longitudinal analysis of T_H_17 cells (CCR4^+^CXCR3^−^CCR6^+^) and **(C)** regulatory T cell (Tregs) with phenotype CD4^+^CD45RO^+^CD25^+^CD127^lo^ in pregnancy and compared with gestation matched controls. Gestation at sampling is indicated in pregnancy (●): <20^+0^ weeks (*N* = *42*), 28 weeks (*N* = *35*), 34 weeks (*N* = *33*), at delivery (*N* = *24*), 24 h post-delivery (*N* = *22*), 6–8 weeks postnatal (*N* = *13*). Non-pregnant controls are depicted as 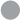 (*N* = *14*). *P* values are two tailed and significance is defined as *P < 0.05 and **P < 0.01 ****P < 0.0001. Non-pregnant and baseline pregnant data analyzed by Mann–Whitney *U* test. Longitudinal data analyzed by generalized linear mixed effects model with gamma log-link and pairwise multiple comparisons of estimated marginal means with sequential Bonferroni correction. Representative flow cytometry plots of **(D)** dendritic cell subtypes mDC and pDC, **(E)** T_H_17 CD4 T cells, and **(F)** CD4^+^ Tregs and, and their respective gating strategies.

**Figure 4:**
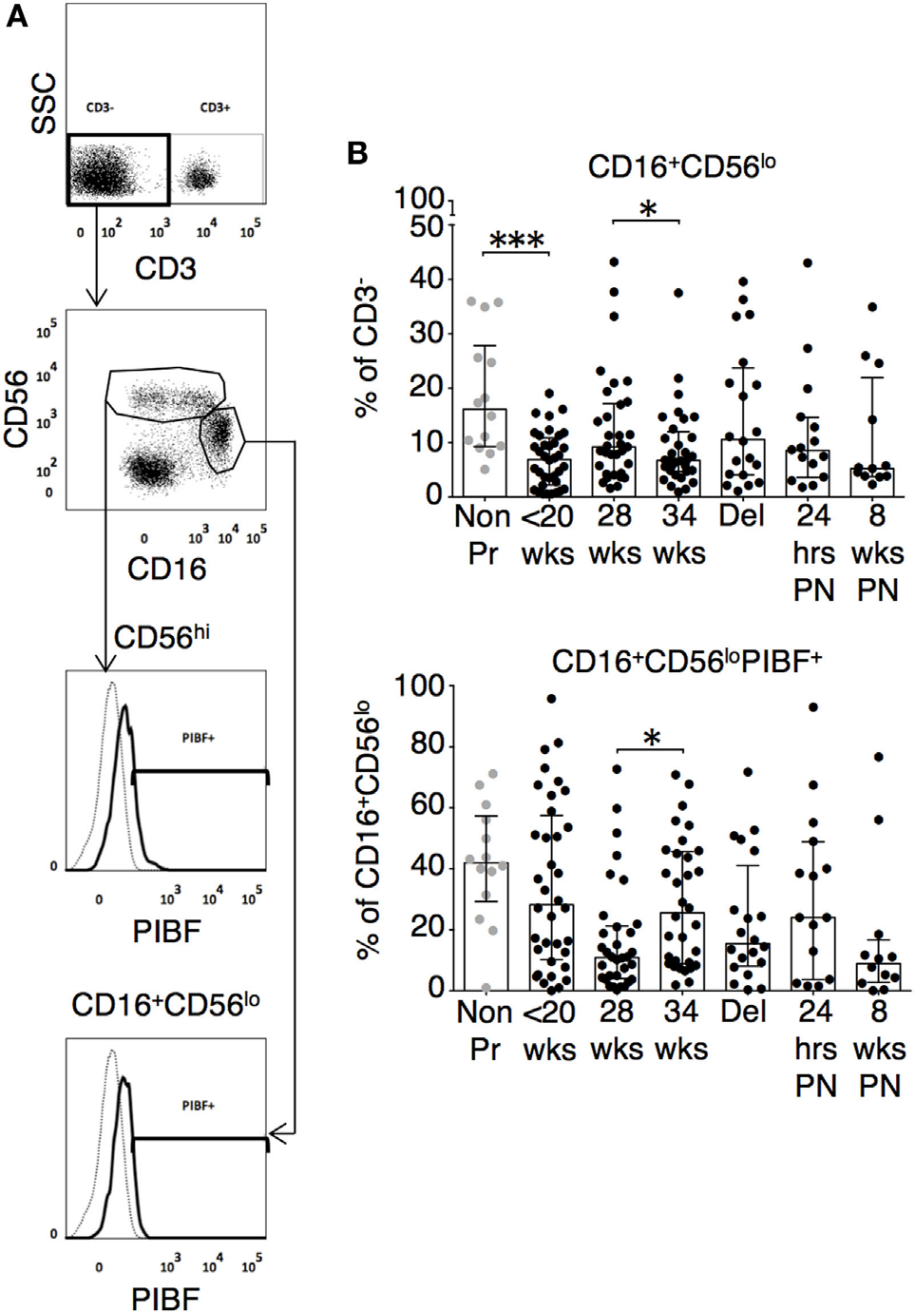
Cytotoxic natural killer (NK) cell subtype with the phenotype CD16^+^CD56^lo^ are reduced and show an increase in P4-induced blocking factor (PIBF) expression in late pregnancy. **(A)** Representative flow cytometry plots are shown for NK cell subsets. **(B)** Longitudinal analysis of CD16^+^CD56^lo^ and their PIBF expression during pregnancy and compared with gestation matched controls. Gestation at sampling is indicated in pregnancy (●): <20^+0^ weeks (*N* = *42*), 28 weeks (*N* = *35*), 34 weeks (*N* = *33*), at delivery (*N* = *24*), 24 h post-delivery (*N* = *22*), 6–8 weeks postnatal (*N* = *13*). Non-pregnant controls are depicted as 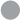 (*N* = *14*). *P* values are two tailed and significance is defined as *P < 0.05 and ***P < 0.001. Non-pregnant and baseline pregnant data analyzed by Mann––Whitney *U* test. Longitudinal data analyzed by generalized linear mixed effects model with gamma log-link and pairwise multiple comparisons of estimated marginal means with sequential Bonferroni correction.

The changes in functional responses were reflected in the leukocyte phenotype observed. CD38 is a surface glycoprotein and enzyme that is important for cell adhesion and regulation of T cell functions including proliferation (31). CD38 expression on CD4 and CD8 T cells was reduced at recruitment and then rose with advancing gestation (Figures 2A,D). This occurred without a similar increase in HLA-DR expression on CD4 or CD8 T cells (not shown). In addition, we observed longitudinal increases in CCR6 expression on CD4 T cells (Figures 2B,D), and increased CD83 expression on both mDC and pDC (Figures 3A,D). CCR6 is important for mucosal immunity. As well as being expressed on T_H_17 cells, the expression of CCR6 on memory T cells enables their migration in response to CCL20 to sites of inflammation (32, 33). Proportions of CD28 expressing effector memory subtypes fell longitudinally during pregnancy (Figures 2C,D). CD28 is an important co-stimulatory marker for T cell activation. However, on memory T cells, the loss of expression correlates with greater peripheral homing and effector function (34). Concurrently, T_H_17 proportions, defined using chemokine markers CCR4 and CCR6 as previously described (35), were reduced in pregnancy compared to non-pregnant controls but saw a longitudinal increase that peaked at 34 weeks (Figures 3B,E). In contrast, CD4 Treg proportions were increased in pregnancy at baseline compared to controls (Figures 3C,F). Collectively, these results were consistent with an initial immune suppression and its subsequent reversal during pregnancy. The perforin/granzyme pathway for target cell killing is used by both cytotoxic T lymphocytes and NK cells (36). Although CD38 expression on CD8 T cells showed a longitudinal increase in pregnancy (Figure 2A), the more cytotoxic NK cell subtype with the phenotype CD16^+^CD56^lo^ (Figure 4A) were reduced and showed an increase in PIBF expression at 34 weeks (Figure 4B). Furthermore, the 9mer FEC peptide pool, which contains CD8 T cell epitopes, predominantly stimulated IL-4 and IL-10 responses (Figure 1A). This suggested that although we demonstrated functionally greater granzyme B responses with gestation, these were concurrently being modulated.

### P4 Suppresses IFN-γ and Granzyme B Responses in Pregnancy

**Figure 5:**
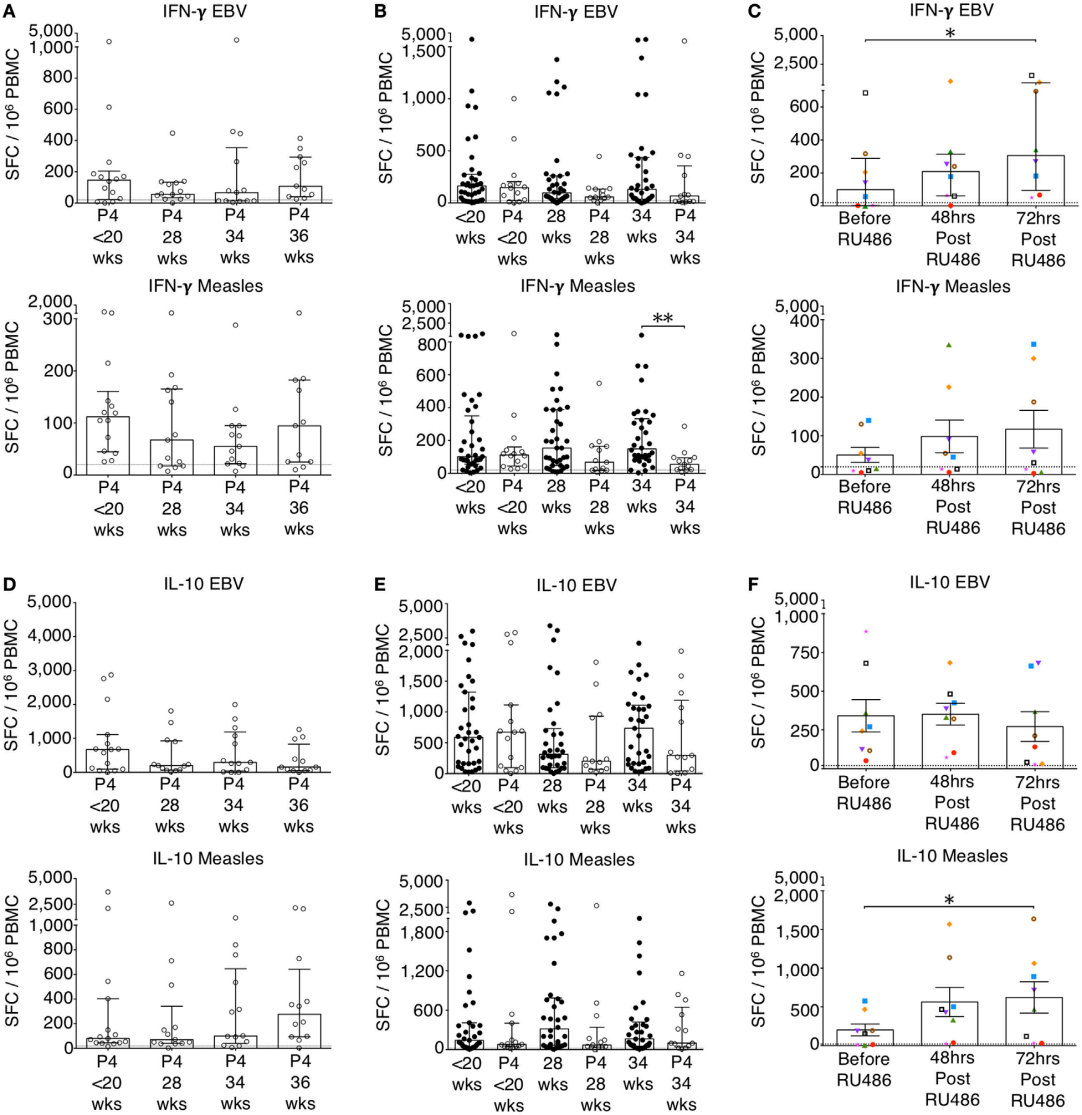
P4 supresses IFN-γ and IL-10 enzyme-linked immunospot (ELISpot) responses. EBV and measles responses are shown. **(A)** Longitudinal analysis of IFN-γ responses in P4 treated. **(B)** IFN-γ responses in P4 treated compared to controls. Unpaired Mann–Whitney *U* test. **(C)** Longitudinal analysis of IFN-γ responses in RU486 treated. **(D)** Longitudinal analysis of IL-10 responses in P4 treated. **(E)** IL-10 responses in P4 treated compared to controls. Unpaired Mann–Whitney *U* test. **(F)** Longitudinal analysis of IL-10 responses in RU486 treated. Gestation at sampling is indicated: in pregnant controls (●) at <20^+0^ weeks (*N* = *42*), 28 weeks (*N* = *35*), 34 weeks (*N* = *33*); and P4 treated pregnancies (⚪) at <20^+0^ weeks (*N* = *15*), 28 weeks (*N* = *13*), 34 weeks (*N* = *13*), 36 weeks (*N* = *11*). For RU486 treated (*N* = *8*) symbols represent individual patients. A dashed line represents <20 spot forming cells/10^6^. Longitudinal data were analyzed with either one-way analysis of variance with Tukey’s *post hoc* correction or Freidman test with Dunn’s *post hoc* correction depending on the data distribution. *P* values are two tailed and significance is defined as *P < 0.05 and **P < 0.01.

**Figure 6:**
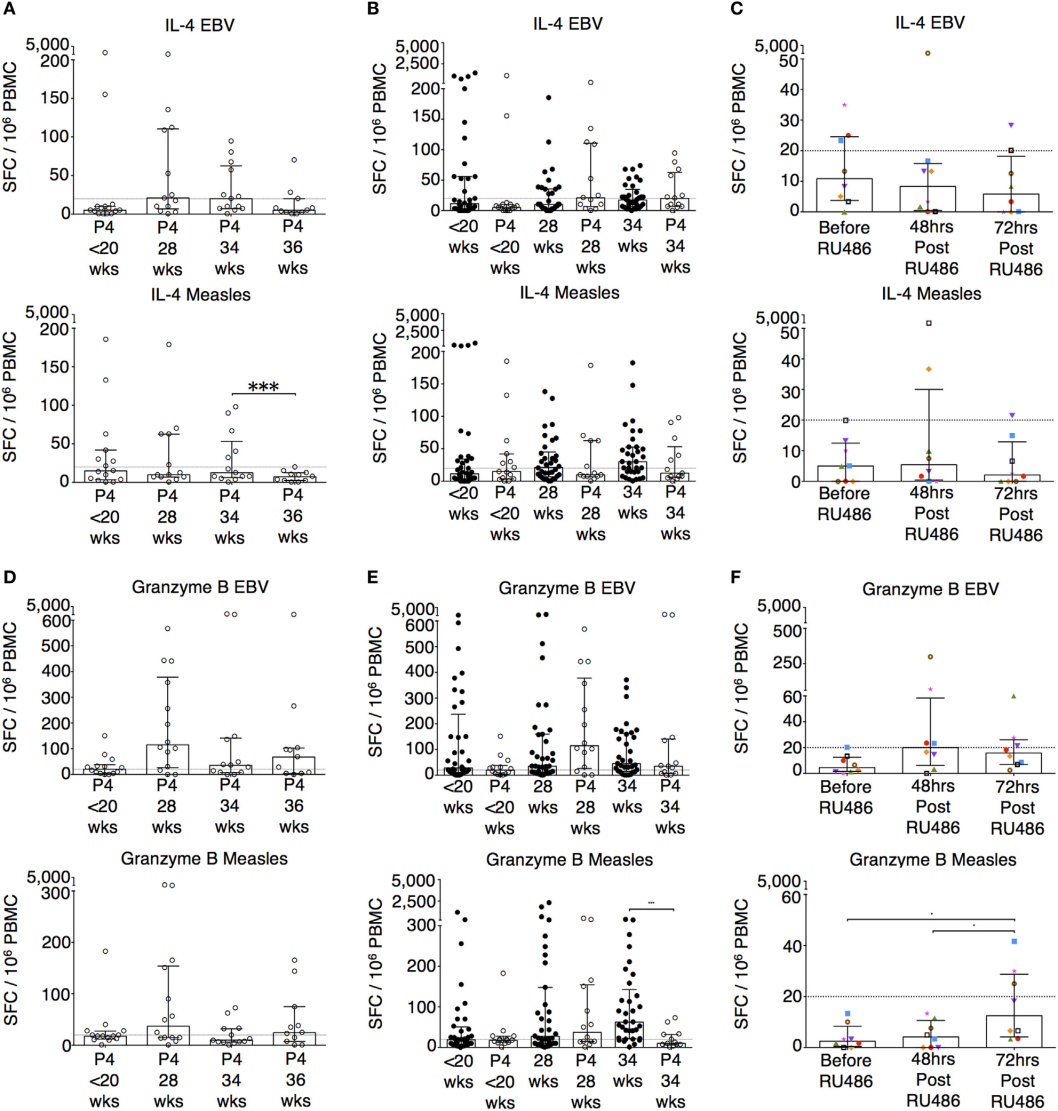
P4 and RU486 have little impact on IL-4 B ELISpot responses but P4 suppresses and RU486 enhances Granzyme B ELISpot responses. EBV and measles responses are shown. **(A)** Longitudinal changes in IL-4 responses with P4 treatment. **(B)** Comparison of IL-4 responses in P4 treated and controls, and **(C)** longitudinal analysis in RU486 treated. **(D)** Longitudinal changes in granzyme B responses with P4 treatment. **(E)** Comparison of granzyme B responses in P4 treated and controls, and **(F)** longitudinal analysis in RU486 treated. Gestation at sampling is indicated: in pregnant controls (●) at <20^+0^ weeks (*N* = *42*), 28 weeks (*N* = *35*), 34 weeks (*N* = *33*); and P4 treated pregnancies (⚪) at <20^+0^ weeks (*N* = *15*), 28 weeks (*N* = *13*), 34 weeks (*N* = *13*), 36 weeks (*N* = *11*). For RU486 treated (*N* = *8*) symbols represent individual patients. A dashed line represents <20 spot forming cells/10^6^. Longitudinal data were analyzed with either one-way analysis of variance with Tukey’s *post hoc* correction or Freidman test with Dunn’s *post hoc* correction depending on the data distribution. *P* values are two tailed and significance is defined as ***P < 0.001.

To determine the effects of P4, we recruited patients receiving either P4 supplementation or P4 antagonism with RU486 for clinically relevant indications as outlined in the Section “Materials and Methods.” Longitudinal differences in IFN-g responses with P4 treatment were not significantly different (Figure 5A). However, when compared to untreated patients, P4 treatment significantly reduced pro-inflammatory IFN-γ responses to measles whole lysate at 34 weeks and TTOX antigen at 28 weeks of gestation (Figure 5B; Figure S2 in Supplementary Material). An opposite effect was seen in the RU486-treated group at 72 h where IFN-γ responses to EBV, influenza A, HSV, measles, and PPD were significantly increased compared to baseline (Figure 5C; Figure S2 in Supplementary Material). Longitudinal and gestation matched analysis of P4 supplemented pregnancies did not show significant changes in antigen-specific IL-10 and IL-4 responses (Figures 5D–F and 6A–C). However, longitudinal analysis of RU486 treated patients showed a significant increase in IL-10 response to measles whole lysate (Figure 5F). Cellular IL-4 production in response to measles whole lysate at 2 weeks post P4 treatment was reduced but remained largely unmeasurable (Figure 6B).

Granzyme B responses showed the same pattern as the IFN-γ responses (Figures 6D,E). P4 treatment was associated with a significantly reduced granzyme B response at 34 weeks of gestation to measles, influenza A and TTOX (Figure 6E; Figure S3 in Supplementary Material). An opposite effect was seen in the RU486 treated group. Although granzyme B production at baseline was below 20 per 10^6^ SFCs, when measurable, PBMC from RU486 treated patients produced a greater number of granzyme B SFCs in response to measles and influenza A whole lysate, and FEC peptide pool measured 72 h post RU486 (Figure 6F; Figure S3 in Supplementary Material).

**Figure 7:**
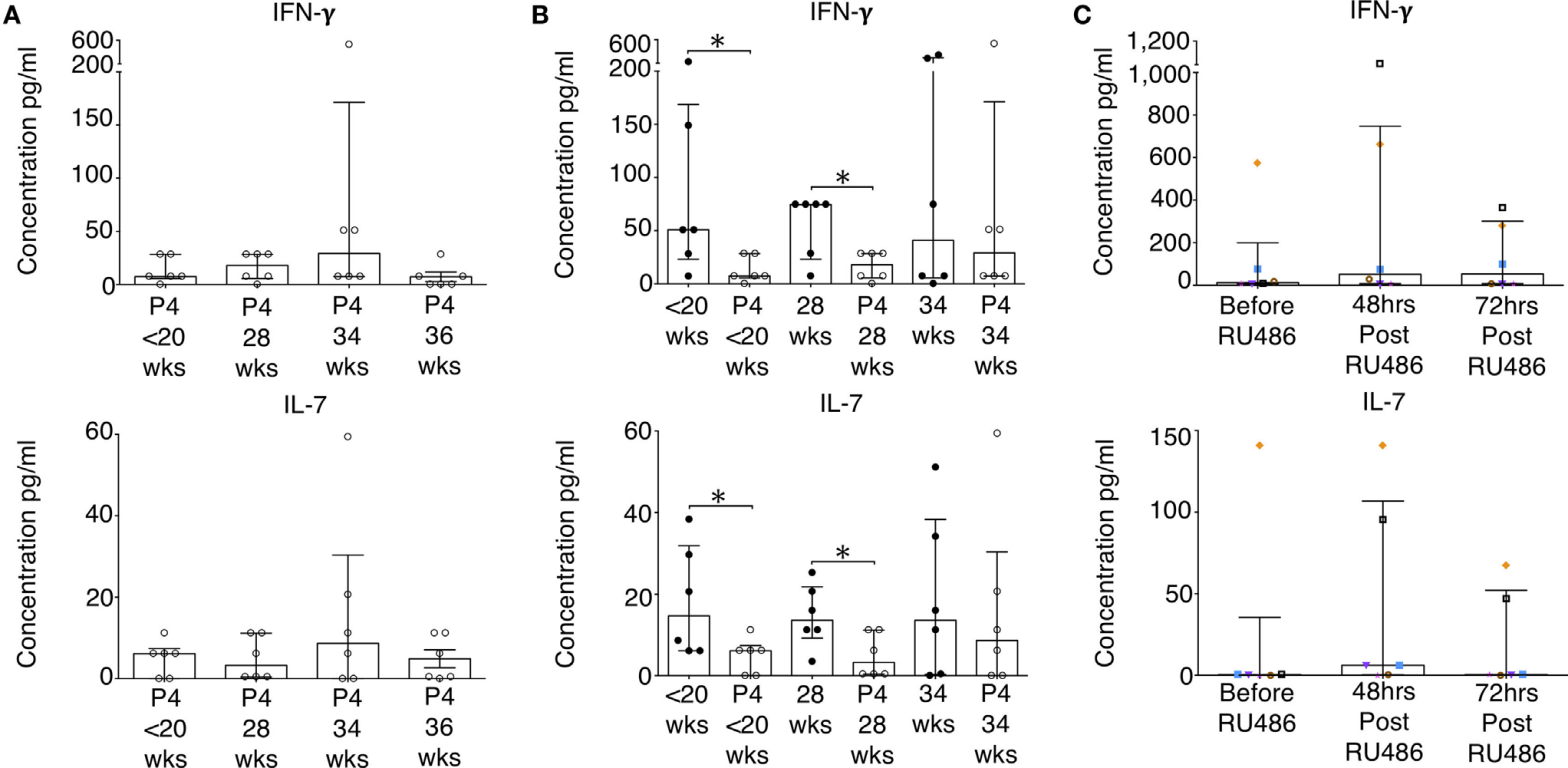
Baseline concentrations of pro-inflammatory cytokines in the P4-treated group are reduced. Plasma concentrations of cytokines measured by 17-plex multiplex assay were determined. **(A)** Longitudinal analysis of IFN-γ and IL-7 concentrations of noted in the P4 treated group. **(B)** IFN-γ and IL-7 concentrations in P4 treated compared to gestation-matched controls. Unpaired Mann–Whitney *U* test. **(C)** Longitudinal analysis of RU486 effect on plasma cytokine concentrations. A representative sample of patients (*N* = *6*) were assessed. Gestation at sampling is indicated: in pregnant controls (●) at <20^+0^ weeks (*N* = *42*), 28 weeks (*N* = *35*), 34 weeks (*N* = *33*); and P4 treated pregnancies (⚪) at <20^+0^ weeks (*N* = *15*), 28 weeks (*N* = *11*), 34 weeks (*N* = *11*), 36 weeks (*N* = *10*). For RU486 treated (*N* = *6*) symbols represent individual patients. Longitudinal data were analyzed with Freidman test with Dunn’s *post hoc* correction. *P* values are two tailed and significance is defined as *P < 0.05.

A representative sample of six patients, from the P4 and RU486 treated groups, with plasma samples taken longitudinally were analyzed to measure cytokine concentrations without cell culture or stimulation. Longitudinally, cytokine concentrations in P4 treated patients remained stable (Figure 7A). However, when compared to gestation-matched controls, baseline IFN-γ, IL-7, and IL-1β were significantly reduced in P4 treated patients (Figure 7B and not shown). This was sustained for IFN-γ and IL-7 at 28 weeks of gestation (Figure 7B). Despite these observations, longitudinal cytokine concentrations in the RU486-treated cohort remained stable (Figure 7C).

### P4 Antagonism Results in Activation of CD4 and Memory T cell Subtypes

**Figure 8:**
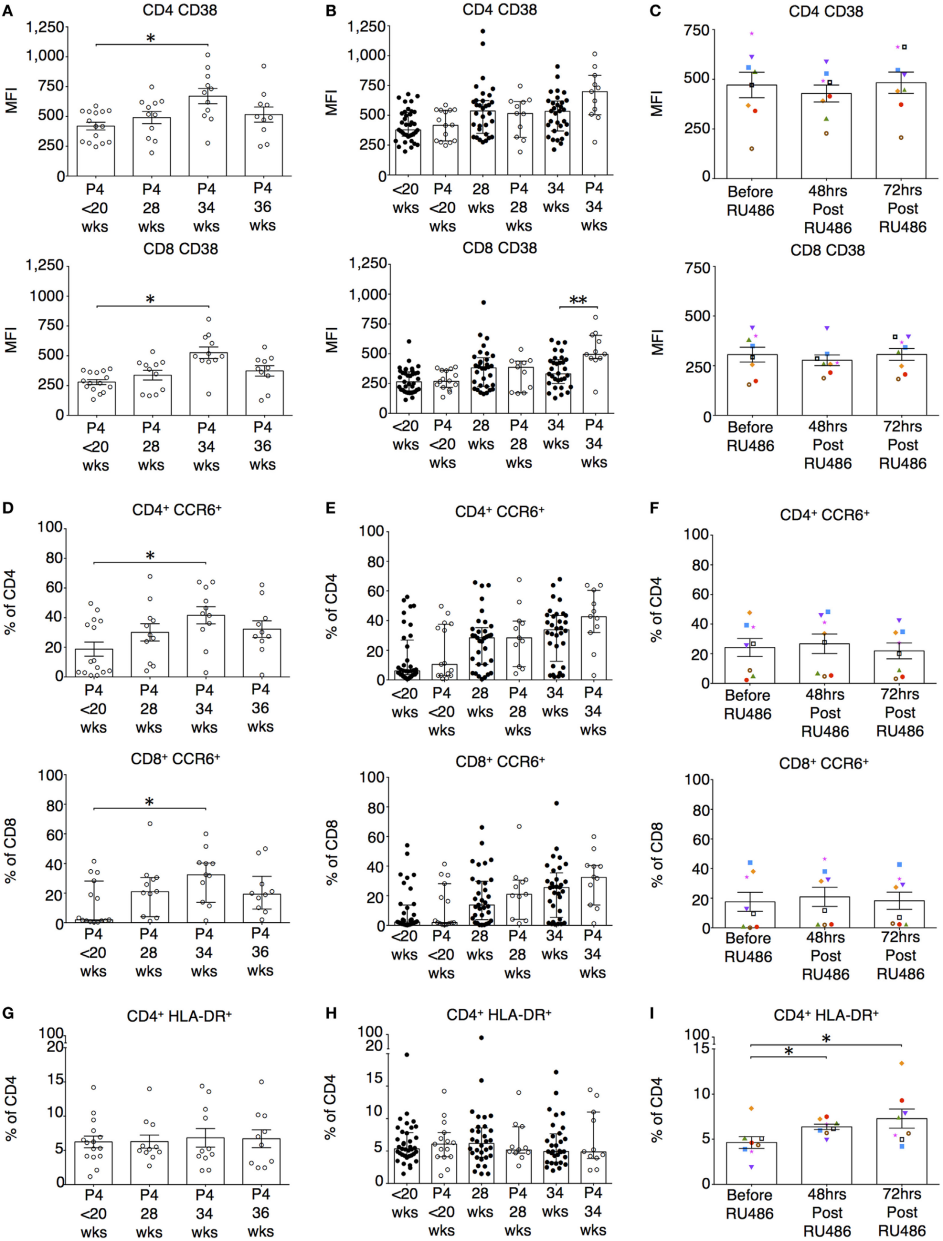
P4 does not suppress CD38 or CCR6 expression on T cells. However, RU486 increases HLA-DR expression on CD4 T cells. **(A)** Longitudinal analysis of CD38 expression measured by mean fluorescence intensity (MFI) on CD4 and CD8 T cells. **(B)** Comparing CD38 expression on CD4 and CD8 T cells in P4 treated with gestation matched controls. Unpaired Mann–Whitney *U* test. **(C)** Longitudinal analysis of the effect of RU486 on CD4 and CD8 T cell CD38 expression. **(D)** Longitudinal analysis of CCR6 expression measured by MFI on CD4 and CD8 T cells (Freidman test with Dunn’s *post hoc* correction). **(E)** Gestation matched comparison of CCR6 expression on CD4 T cells between P4 treated and controls. **(F)** Longitudinal analysis of CCR6 expression on CD4 T cells from the RU486 group. **(G)** Longitudinal variation in HLA-DR expression on CD4 T cells in the P4-treated group. **(H)** Gestation-matched comparison of HLA-DR expression on CD4 T cells between P4 treated and controls. **(I)** Longitudinal analysis of HLA-DR expression on CD4 T cells from the RU486 group. Gestation at sampling is indicated: in pregnant controls (●) at <20^+0^ weeks (*N* = *42*), 28 weeks (*N* = *35*), 34 weeks (*N* = *33*); and P4 treated pregnancies (⚪) at <20^+0^ weeks (*N* = *15*), 28 weeks (*N* = *11*), 34 weeks (*N* = *11*), 36 weeks (*N* = *10*). For RU486 treated (*N* = *8*) symbols represent individual patients. Longitudinal data was analyzed with either one-way analysis of variance with Tukey’s *post hoc* correction or Freidman test with Dunn’s *post hoc* correction depending on the data distribution. *P* values are two tailed and significance is defined as *P < 0.05 and **P < 0.01.

The longitudinal changes seen in the expression of CD38 and CCR6 on both CD4 and CD8 T cells in the un-supplemented group were reproduced in the P4 treated (Figures 8A,D). P4 had no impact on CD38 or CCR6 expression on CD4 T cells when compared to gestation-matched controls (Figures 8B,E). However, with P4 supplementation, CD38 expression on CD8 T cells at 34 weeks of gestation was increased (Figure 8B). Nonetheless, CD38 and CCR6 expression was unaffected by RU486 (Figure 8C,F). Supplemental P4 appeared to have no impact on CD4 and CD8 HLA-DR expression either longitudinally nor when compared to gestation-matched controls (Figures 8G,H and data not shown). However, antagonism with RU486 resulted in increased expression of HLA-DR on CD4 but not on CD8 T cells (Figure 8I and data not shown).

**Figure 9:**
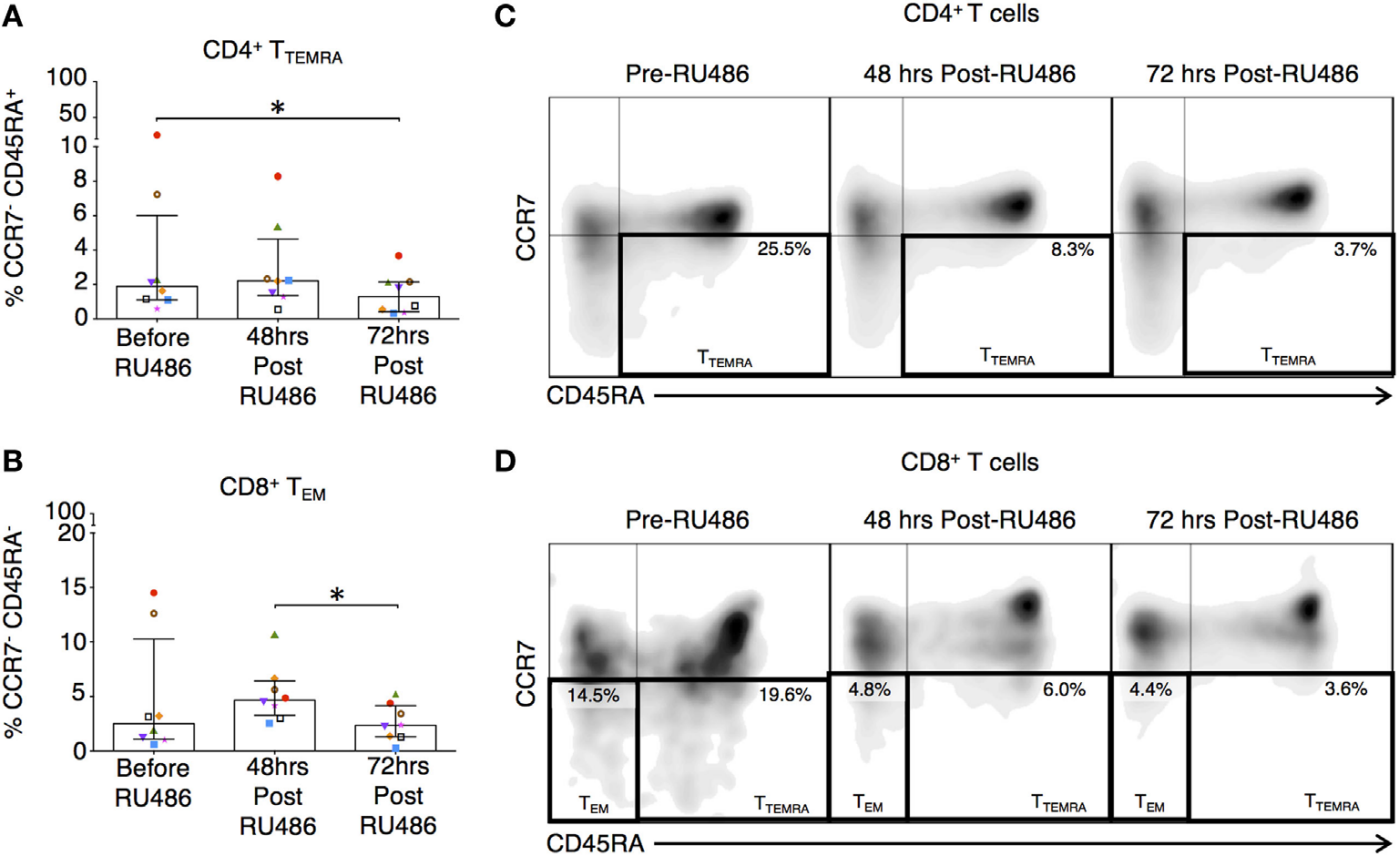
RU486 treatment is associated with reduced proportions of CD4 and CD8 differentiated effector memory subtypes. **(A)** Longitudinal analysis of proportions of CD4^+^CCR7^−^CD45RA^−^ T_EM_ and **(B)** CD8 T_EM_ (CCR7^−^CD45RA^−^) in RU486-treated (*N* = *8*) symbols represent individual patients. *P* values are two tailed and significance is defined as *P < 0.05, by Freidman test with Dunn’s *post hoc* correction. Representative flow cytometry plots of **(C)** singlet CD4^+^ lymphocytes co-expressing CCR7 and CD45RA is shown with percentages of T_EM_ cell, and **(D)** singlet CD8^+^ lymphocytes co-expressing CCR7 and CD45RA is shown with percentages of T_EM_ and T_TEMRA_ cells. Populations were gated using isotype controls.

**Figure 10:**
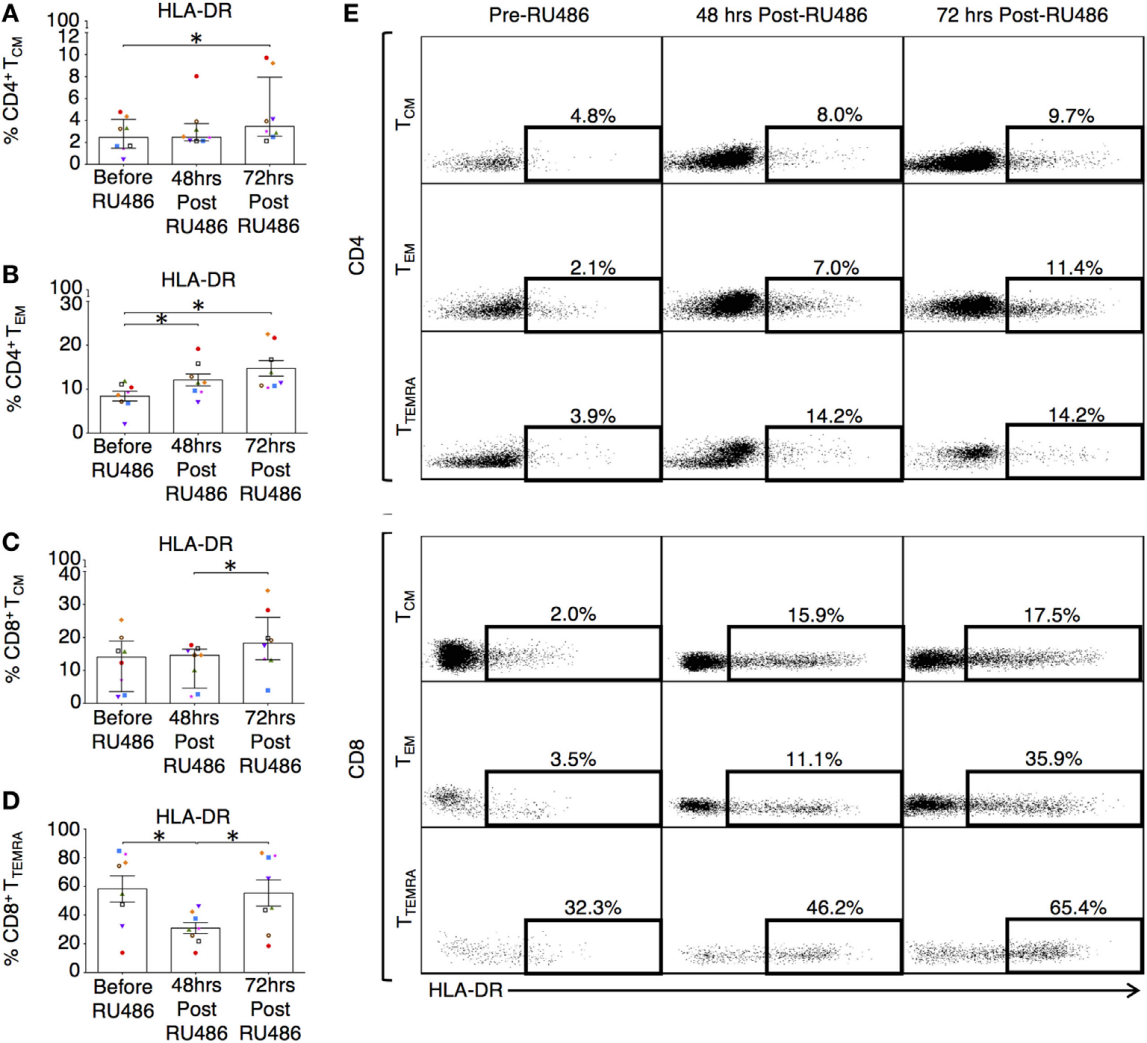
Differentiated effector memory subtypes in RU486 treated have an increased expression of HLA-DR. **(A)** Longitudinal change in percentage expression of HLA-DR on: CD4 T_CM_ (Freidman test with Dunn’s *post hoc* correction), **(B)** CD4 T_EM_ [one-way analysis of variance (ANOVA) with Tukey’s *post hoc* correction], **(C)** CD8 T_CM_ (Freidman test with Dunn’s *post hoc* correction), and **(D)** CD8 T_TEMRA_ (one-way ANOVA with Tukey’s *post hoc* correction). *P* values are two tailed and significance is defined as *P < 0.05. **(E)** Representative flow cytometry plots showing the percentage expression of HLA-DR on both CD4 and CD8 (CCR7^+^CD45RA^−^) T_CM_, (CCR7^−^CD45RA^−^) T_EM_, and (CCR7^−^CD45RA^+^) T_TEMRA_ subsets in PBMC from subjects receiving RU486 treatment. Memory CD4^+^ and CD8^+^ subset HLA-DR expression, gated with SSC on the vertical axis is shown. Populations were gated using isotype controls and percentages of HLA-DR expressing cells is indicated.

Memory T cells provide quantitatively enhanced responses to secondary or recall antigen challenge. In our study, these subsets were defined as previously described (37). Their activated and expansive potential was determined by their expression of HLA-DR and CD28 as these are associated with peptide presentation and co-stimulation (34). Proportions of CD4 terminally differentiated effector memory (T_TEMRA_) T cells and CD8 T_EM_ and T_TEMRA_ were reduced 72 h post RU486 treatment (Figures 9A–E). CD4 T_CM_ expressing HLA-DR and CD28 (Figures 10A,E and not shown) showed an increase 72 h post RU486 and proportions of CD4 T_EM_ expressing HLA-DR increased 48 h following RU486 (Figures 10B,E). HLA-DR^+^ CD8 T_CM_ proportions increased 72 h post RU486 but HLA-DR^+^ CD8 T_TEMRA_ decreased 48 h post RU486 and returned to baseline 72 h post RU486 (Figures 10C–E).

### P4 Was Associated With a Fall in Proportions of Tregs, but Had No Impact on T-Helper Subtypes or PIBF-Expressing T Cells

**Figure 11:**
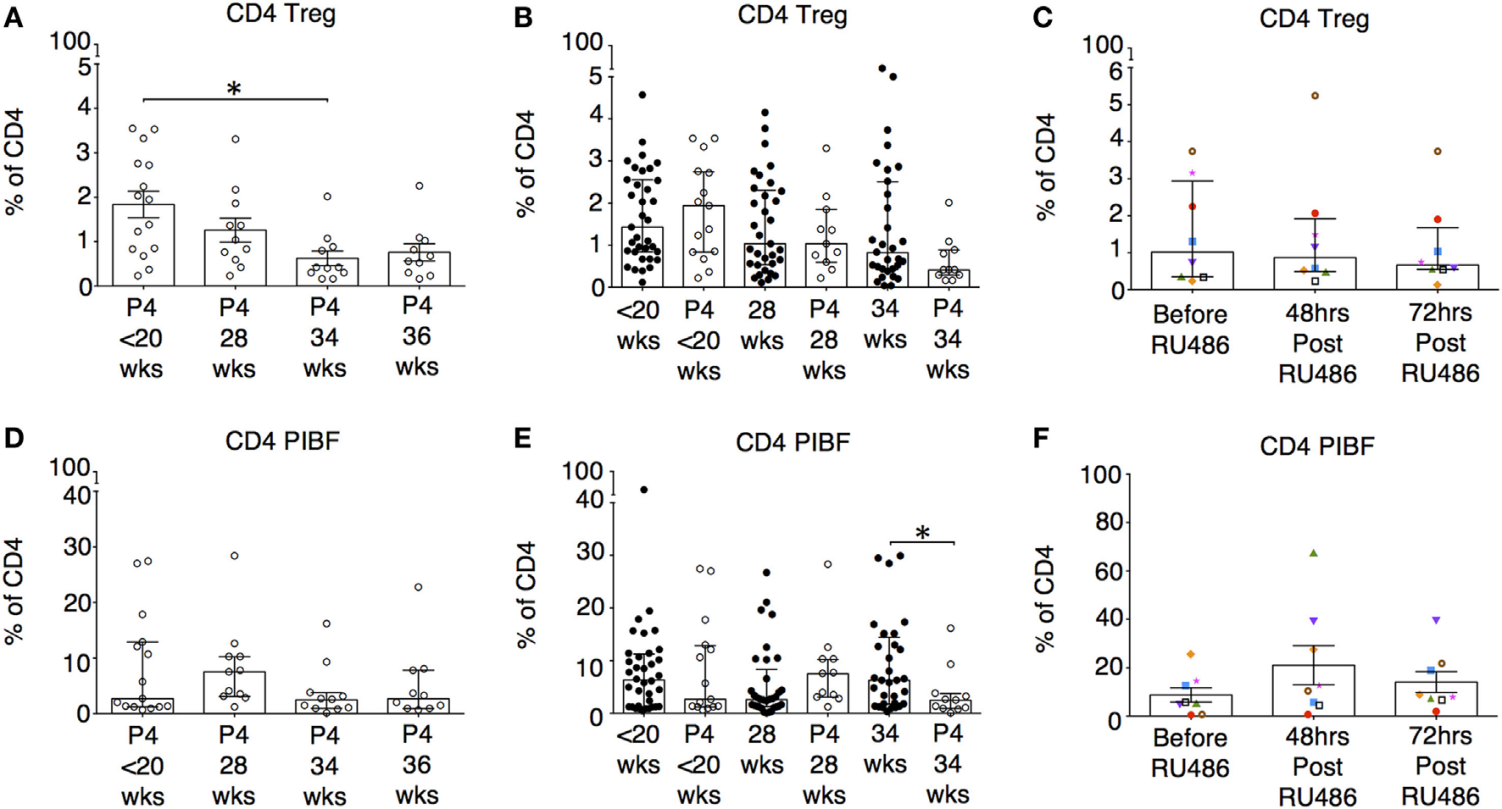
The influence of P4 and RU486 on CD4^+^ regulatory T cell (Tregs) and P4-induced blocking factor (PIBF) expressing CD4^+^ T cells. **(A)** Longitudinal analysis of CD4 Treg proportions (CD4^+^CD45RO^+^CD25^+^CD127^lo^) in peripheral blood obtained from P4 treated patients. **(B)** Gestation-matched paired comparisons of Treg proportions in peripheral blood obtained from P4 treated versus untreated pregnant controls that were previously analyzed longitudinally in Figure 3C. **(C)** Longitudinal analysis of Treg proportions in peripheral blood obtained from RU486 treated pregnant patients. **(D)** Longitudinal analysis of PIBF expressing CD4 T cells in peripheral blood obtained from P4-treated patients. **(E)** Gestation matched paired comparisons of PIBF expressing CD4 T cells in peripheral blood obtained from P4 treated versus untreated pregnant controls (Unpaired Mann–Whitney *U* test). **(F)** Longitudinal analysis of PIBF expressing CD4 T cells in peripheral blood obtained from RU486-treated pregnant patients. Gestation at sampling is indicated: in pregnant controls (●) at < 20^+0^ weeks (*N* = *42*), 28 weeks (*N* = *35*), 34 weeks (*N* = *33*); and P4 treated pregnancies (′) at <20^+0^ weeks (*N* = *15*), 28 weeks (*N* = *11*), 34 weeks (*N* = *11*), 36 weeks (*N* = *10*). For RU486 treated (*N* = *8*) symbols represent individual patients. Longitudinal data were analyzed with either one-way ANOVA with Tukey’s *post hoc* correction or Freidman test with Dunn’s *post hoc* correction depending on the data distribution. *P* values are two tailed and significance is defined as *P < 0.05.

Although P4 has previously been shown to be able to generate induced Tregs *in vitro*, in our study, proportions of CD4 Tregs decreased longitudinally with P4 treatment (Figure 11A). However, when proportions of Tregs with P4 supplementation were compared to gestation matched controls, there were no differences (Figure 11B). In addition, the longitudinal P4 effects were not reciprocated with the use of RU486 (Figure 11C). This led us to assume *in vivo*, P4 supplementation has little effect on the induction of Tregs in the periphery. Similarly, proportions of T-helper subtypes T_H_1, T_H_2, and T_H_17 were unaffected by the use of P4 or RU486 (not shown). There were no longitudinal effects of P4 supplementation on CD4 PIBF expression. However, when compared to controls (Figure 11D), PIBF expression on CD4 T cells was decreased at 34 weeks of gestation, but the reverse was not observed with RU486 treatment (Figures 11E,F). PIBF expression on CD3 γδ or CD8 T cells was unaffected by P4 or RU486 (not shown).

### P4 Does Not Appear to Affect NK or DC Phenotype

Overall, P4 had no longitudinal effects on NK cell expression of PIBF (Figures S4A,D,E in Supplementary Material). CD16^+^CD56^lo^ NK cells expressing PIBF represented a smaller proportion when compared to un-supplemented pregnancies at 34 weeks (Figure S4B in Supplementary Material). However, RU486 had no effect (Figure S4C in Supplementary Material). Likewise, proportions of CD56^hi^ and iNKT did not change with P4 supplementation or RU486 treatment (Figures S4E,F,H,I in Supplementary Material).

Despite the previously mentioned changes seen on memory T cells, proportions of activated mDC and pDC expressing co-stimulatory markers, and HLA-G expressing CD14^+^ DC-10 (38) remained un-affected by P4 or RU486 (not shown).

## Discussion

In this study, we investigated the effect of P4 on the peripheral maternal immune system. We used a novel approach by recruiting pregnant women taking P4 or RU486 using clinically effective regimens specifically designed to either reduce their risk of preterm labor or induce a functional P4 withdrawal with the aim of initiating labor. Furthermore, we are the first group to examine the *in vivo* effects of RU486 on leukocyte function in the mid second trimester of pregnancy. Our data demonstrate that, in pregnancy, P4 reduces both pro-inflammatory and cytotoxic T cell responses, and that this suppressive effect is reversed with the use of RU486.

We have previously shown that, in normal pregnancy, IFN-γ and IL-10 responses to recall antigens are elevated in the third trimester (39). This occurs despite an increase in CD4 T_EM_ in the second trimester, suggesting that IL-10 may restrict the activation of the immune system by suppressing antigen specific T cell responses (39, 40). In the current study, our findings supported this concept. Both IFN-γ and IL-10 peripheral recall responses were largely elevated at delivery. Notably though, IL-10 responses showed marked increases in pregnancy across gestation despite a leukocyte phenotype that seemed to suggest a gradual decline of IL-10 antigen-induced immune tolerance with a longitudinal increase in T_H_17 proportions, and the loss of any baseline pregnancy-related bias in CD4 Treg proportions. This occurred alongside an increasingly activated immune system, suggested by longitudinal increases in activated (CD38) and migratory (CCR6) T cells, mature memory T cells (CD28) with greater homing and effector function (34), and activated DCs (CD83). This gradual decline of immune-modulation was also evident in humoral and cytotoxic responses suggested by more frequent IL-4 SFCs in pregnancy, as well as increased granzyme B cytotoxic T cell activity, and IFN-γ and IL-10 responses to CD8 epitopes at delivery. In contrast, other studies have suggested that both humoral and cell-mediated immune responses are largely reduced in the third trimester, but innate responses to bacteria are increased (20, 41, 42). Our IL-4 findings likely reflect heightened humoral immune function in pregnancy, but the change in cytotoxic responses are suggestive of a loss of modulation of antigen specific memory CTL functions. We sought to determine how influential P4 may be as a driver of these changes.

Of note in our non-pregnant controls, we did not control for different phases in their menstrual cycle. In non-human primates, in peripheral blood, the luteal phase of the menstrual cycle, when P4 is at peak concentration, is associated with tolerant immune responses that favor successful pregnancy (43). It is assumed these changes transfer to humans. However, the majority of studies investigating the effects of menstruation show conflicting data. For example, although some researchers have shown Tregs initially expand during the follicular phase and then decrease mid-luteal, and that the luteal phase is associated with a decline in PBMC proliferation and IFN-γ production, T cell PHA responses are unchanged, and variations in Th1/Th2 cytokines have not been shown consistently in the literature (44–49). In contrast, NK cytotoxicity may be influenced by menstruation (44, 50). It is also important to appreciate that previous exposure to paternal antigens induces tolerance and so parity and sexual activity may enhance the effects of P4 (45, 51, 52). Furthermore, when compared to pregnant patients, the peak serum P4 concentration in non-pregnant women is approximately 15 versus 60 ng/ml in early and 180 ng/ml in late pregnancy, which represent a 4- and 12-fold increase, respectively (53, 54). This suggests that any changes due to menstrual variation in serum P4 are unlikely to compare to changes in pregnancy. In fact, in our previous work, we found that despite *in vitro* culture with P4, leukocyte PIBF expression in controls, at different stages in their menstrual cycle, did not equal those seen in pregnancy suggesting that neuro-endocrine differences are not the sole determinant of leukocyte phenotype in pregnancy (39). Therefore, in our study, peripheral blood samples were not controlled for phases of the menstrual cycle.

Serum concentrations of endogenous P4 increase throughout pregnancy and reach 175–636 nmol/l in the maternal circulation during the third trimester (53). Yet, as a potent endogenous suppressor of cytotoxic immune responses and regulator of cytokine secretion, our results suggest its effects decline after 34 weeks of pregnancy. Therefore, we expected the addition of exogenous P4, as well as modeling its withdrawal using RU486, would be noticeable until the end of the second trimester pregnancy and thereafter become less obvious. Previous studies have shown that, outside of pregnancy, P4 is antiproliferative and suppresses the production of the pro-inflammatory cytokines IFN-γ, TNF-α, while upregulating IL-4 in PBMC cultures with PHA, and that these effects are reversed by RU486 (1, 14). In addition, P4 has been shown to suppress cytotoxic T cell activity (12). Conversely, RU486 has been shown to increase uterine NK cytotoxicity (12, 55, 56). In human and animal models, P4 also has systemic and tissue specific anti-inflammatory effects, acting predominantly *via* the inhibition of NFκB and MAPK/AP-1 pathways (3, 57–59). However, in some disease states, P4 is less effective once pro-inflammatory processes are established, and this may be associated with a differential tissue expression of PR isoforms (60, 61). In fact, in myometrial tissue, this is thought to be one of the contributors to the onset of term labor (62). We found that, in contrast to control pregnancies, P4 supplementation appeared to encourage a stable IFN-γ response that was significantly reduced when compared to gestation-matched controls. This occurred without any negative effect on longitudinal CD38 and CCR6 T-cell expression, which continued to increase despite exogenous P4 supplementation. Both receptors are important for inflammatory responses and cell migration. CD38 is characteristically raised in chronic inflammation and is associated with an enhanced ability to produce IFN-γ (63, 64). CCR6 is expressed on effector memory populations that contribute to recall responses (33). RU486 treatment had the opposite effect, with significant increases in IFN-γ responses posttreatment. These changes were statistically significant 72 h post RU486, which was post-delivery, rather than 48 h post treatment. However, altogether, our findings point to a P4 mediated effect rather than a consequence of delivery. P4 is known to negatively effect polyfunctional cytokine production from CD8 T cells and suppresses decidual lymphocyte cytotoxicity (12, 14). Granzyme B is primarily produced by activated CTLs and NKs cells and is, therefore, a useful surrogate marker for the functional activity of CTLs. Similar to the IFN-γ data, granzyme responses in the P4 treated group, when compared to gestation-matched controls at 34 weeks of pregnancy, were reduced; conversely, in the RU486 treated group, the response was increased, and these included responses against FEC peptide pool that incorperates CD8 specific epitopes. Although the latter results were significant post delivery, the control pregnancies did not show delivery was associated with any changes in the granzyme B response, suggesting that the differences are are most likely to be RU486 related.

Interestingly, although IL-10 and IL-4 responses in the P4 as well as in the RU486 treated groups were, on the whole, unaffected, suggesting the altered antigen-specific responses are not T_H_2 or IL-10 driven. Our findings contradict murine models that have previously shown that in pregnancy, systemic and uterine Treg proportions as well as their suppressive activity is increased with P4 and blocked by RU486, but this increase does not necessarily prevent spontaneous fetal loss (65). Schumacher et al. showed that in abortion-prone mice, although decidual Treg numbers increased following intra-peritoneal P4 administration, this did not result in greater fetal survival (66). It is possible that these Tregs may have been poorly functioning or that other pathways are more influential at improving fetal outcomes. Of note, the human equivalent doses (HED) of P4 used in both of these studies were either comparable or lower than the 400 mg P4 dose used in our study, and the RU486 HED (30 mg) used by Mao et al. was far less than that used in our study (200 mg) (65–67). Therefore, the different findings in their work compared to ours are surprising. All of the aforementioned murine studies analyzed the effects of P4 in early and mid pregnancy in mice, when the maternal response to the initial surge in fetal antigen is vital for ongoing pregnancy success. Therefore, expansion of potentially poorly functioning Tregs may not be sufficient to improve tolerance to fetal antigen. Interestingly, intravenous adoptive transfer of Tregs from normal pregnant to abortion prone mice at days 0–4 of pregnancy improves placentation and reduces reabsorption rates (68–70). However, miscarriage rates were not completely abrogated in these studies, and the same positive findings were not reproduced when the Tregs were isolated from non-pregnant mice (68–70). Our own results in pregnancy compared to non-pregnant controls showed an increased proportion of Tregs at baseline. However, we also found a longitudinal fall in Treg proportions with P4 supplementation. Mjösberg et al. have previously predicted such an effect, where, *in vitro*, P4 and 17β-estradiol reduced functionally suppressive CD4 Foxp3^+^ Tregs (71). Unfortunately, we did not investigate the suppressive activity of Tregs in this study, but the fall in number likely reflects the systemic anti-inflammatory effect of P4 and suggests pathways other than those involving Tregs are at play.

It is also worth noting that our unstimulated sera concentrations of IFN-γ, IL-1β, and IL-7 were significantly reduced pretreatment in the P4 group, and IFN-γ and IL-7 were also comparatively reduced at 28 weeks, suggesting that the effects were P4 related. These findings were not reciprocated in the RU486 group. The most likely confounder is that the P4-treated cohort represents patients at risk of premature delivery and so may have inherent immune differences. However, there were no other baseline differences between groups despite two of the patients delivering preterm at 34+ weeks of gestation immediately after stopping P4 treatment.

Our ELISpot results suggest that P4 may disrupt pro-inflammatory cell-mediated responses and its prolonged use is associated with suppressed antigen specific memory CTL responses, which are not fetal antigen specific. From our data, the latter effect does not seem to be driven by PIBF unlike in previous published reports (12, 72). In the P4-treated group, despite proportions of PIBF expressing CD4 T cells and cytotoxic CD16^+^CD56^lo^ NK cells being reduced at 34 weeks of gestation, our RU486 data did not show any reciprocal changes. This is unusual as P4 has been shown to promote the production of PIBF from lymphocytes *in vitro via* PR (72). PIBF has a known anti-cytotoxic effect on NK cells, and this is thought to be by co-localizing in cytoplasmic granules and blocking degranulation (73, 74). Therefore, our results suggest that P4 supplementation in pregnancy has a limited effect on cellular PIBF expression, and consequently, that its effect on gestational length is independent of PIBF synthesis. It is likely that this is because P4 is acting *via* GR and thus the lack of opposite effects by RU486 may be due to different receptor-binding affinity (75, 76). Furthermore, GR binding may not always produce an immune suppressed affect. In transgenic rats, endogenous GR engagement is associated with an increase in activated and memory T cells (77). Regardless, our results showed suppressed IFN-γ responses, but we did not find any differences in memory T cell activation marker expression. This is in contrast to *in vitro* studies in animals, which have shown that in addition to inhibiting DC stimulation of naїve T cells in rats and memory CD8 IFN-γ production in mice, P4 reduces murine memory CD8 T cells during heterosubtypic influenza virus challenge (78–80). However, in the RU486 group, we found changes in MHC class II molecule expression on memory T cells that was highly suggestive of a reversal of pregnancy associated immune-modulation. The expression of HLA-DR increased on CD4 T_EM_ cells post RU486 treatment, and although the expression of HLA-DR was reduced on CD8 T_TEMRA_ cells, it subsequently increased 72 h post RU486. It is possible that CD8 T cells have a greater threshold for antigen exposure, and that the contributions of T_EM_ and T_CM_ cells can vary (81, 82). Furthermore, we show delivery in the presence of RU486 was associated with a fall in T_TEMRA_ and T_EM_ proportions, but with concurrent increases in HLA-DR expression. Surface MHC class II molecule expression on CD4 T cells corresponds to an improved ability for these cells to present antigen and regulate immune responses (83). Previously, murine data suggested that RU486 inhibits apoptosis of mature DC *via* GR and promotes HLA-DR expression on monocytes in humans (84, 85). Therefore, the effects of RU486 may be to initiate a rapid recovery of immune function as demonstrated by the ELISpot data, since, classically, memory T cells are highly responsive to recall antigens.

In conclusion, this study describes a novel method of examining the effects of P4 on the maternal immune system. Our data suggest that exogenous P4 reduces pro-inflammatory and cytotoxic T cell responses in pregnancy. It achieves this by a combination of effects on cell-mediated interactions, including altering memory T cell antigen sensitivity and regulating leukocyte migration. These effects are, in part, reversed with the use of RU486. Our results have identified which aspects of the maternal immune response are P4-regulated, as such modulation of these pathways may have potential as future therapeutic targets with the aim of modulating the maternal immune response to pregnancy. Future *in vivo* human work will help to key establish the cellular interactions at play during human pregnancy.

## Ethics Statement

All subjects were recruited from Chelsea and Westminster Hospital, London, UK. This study was carried out in accordance with the recommendations of National Institute of Health Research (NIHR) Good Clinical Practise guidelines, and a NHS Research Ethics Committee. The protocol was approved by the National Research Ethics Service (NRES), London, UK committee as well as by Chelsea and Westminster NHS Trust, London, UK; Ref: 11/LO/0971. All subjects gave written informed consent in accordance with the Declaration of Helsinki.

## Author Contributions

NS, NI, and MJ had a substantial contribution to the conception and design of the project and its interpretation; were responsible for the acquisition, analysis, and interpretation of the data, and drafted the work. All authors contributed to revising of the manuscript and have approved the final version. All authors agreed to be accountable for all aspects of the work in ensuring that questions related to the accuracy or integrity of any part of the work are appropriately investigated and resolved.

## Conflict of Interest Statement

The authors declare that the research was conducted in the absence of any commercial or financial relationships that could be construed as a potential conflict of interest.
