# Supplementary material for: Progesterone Modulation of Pregnancy-Related Immune Responses
Source: Front Immunol. 2018 Jun 20;9:1293. doi: 10.3389/fimmu.2018.01293 (PMC6020784; doi:10.3389/fimmu.2018.01293)
Supplement: Supplementary file 1 [file Presentation_1.PDF]

frontiers

Supplementary Material

Progesterone Modulation of Pregnancy-Related Immune Responses

Nishel M Shah, Alex Cocker, Nesrina Imami and Mark R Johnson*

*** Correspondence:** Professor Mark R Johnson: mark.johnson@imperial.ac.uk

1 Supplementary Tables and Figures

1.1 Table S1 Demographic Details of each of the Patient Groups in the Study

	Controls (HC)	Longitudinal Pregnancy (Pr)	P4 Treated (P4)	RU486 Treated	HC vs Pr	Pr vs P4
**Ethnicity**					NS	P=0.0201
Caucasian	72%	89%	56%	73%		
Black	14%	2%	25%	0%		
Asian	7%	7%	13%	27%		
Mixed	7%	2%	6%	0%		
**BMI** (kg/m^2)		23<br>IQR 21-25	24<br>SD ±3.3	25<br>SD ±5.0		NS
**Age** (completed Years)	25.5<br>IQR 24.0-29.5	33.7<br>SD ±3.4	34.3<br>SD ±2.7		P=0.0002	P=0.6119
**Parity**					NS	P<0.0001
0	86%	67%	13%	45%		
1	14%	33%	63%	55%		
2	0%	0%	25%	0%		
**IOL**						NS
Yes		26%	13%			
No		74%	87%			
**Birth weight** (g)		3219<br>SD ±434	3057<br>SD ±163			NS
**Mode of delivery**						NS
Elective LSCS		18%	25%			
Emergency LSCS		18%	19%			
SVD		31%	44%			
Instrumental		33%	13%			
**Early miscarriages** (12 weeks)						
0		74%	69%	64%		
1		26%	19%	27%		
>1		0%	13%	9%		
**Late miscarriages** (16-24 weeks)						
0		100%	94%	100%		
1		0%	6%	0%		
>1		0%	0%	0%		
**Gestation** (median) at delivery as completed weeks		39<br>IQR 38-40	38<br>IQR 35-39	19<br>SD ±1.8		P=0.0013

Related to experimental procedures, subjects. Demographic data for: non-pregnant controls (HC), pregnant controls (Pr), progesterone supplemented (P4) and RU486 treated. Grouped data is represented as percentages, and continuous data as mean and SD or median and IQR depending on the data distribution. Statistical analysis was undertaken using Student's T test or Mann-Whitney U for continuous data, and Fisher's exact test was used for grouped variables.

1.2 Figures

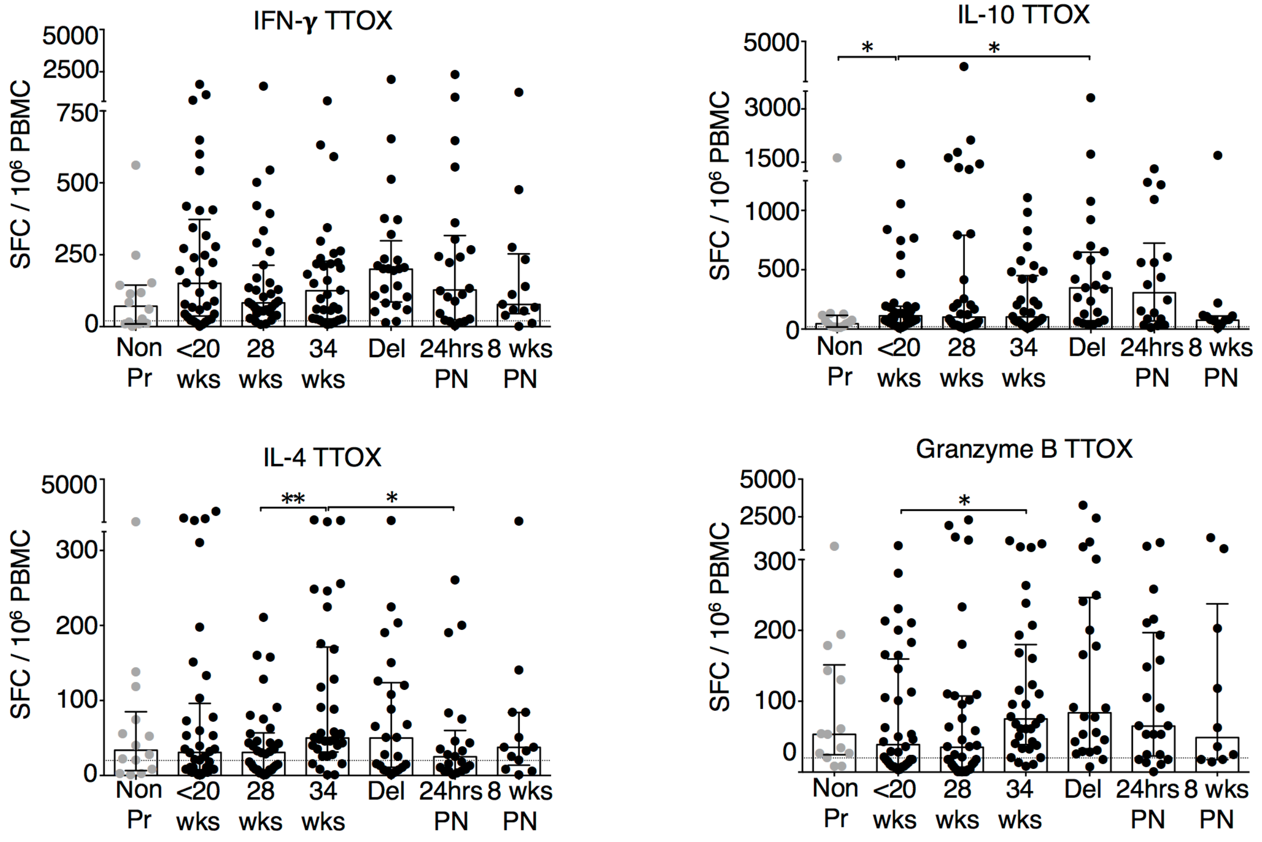

Figure S1. Graphical representation of the IFN-γ, IL-10, IL-4 and Granzyme B ELISpot responses to TTOX antigen. Columns indicate median and IQR. Gestation at sampling is indicated in pregnancy (●): <20^{+0} weeks (*N=42*), 28 weeks (*N=35*), 34 weeks (*N=33*), at delivery (*N=24*), 24 hours post-delivery (*N=22*), 6-8 weeks postnatal (*N=13*). Non-pregnant controls are depicted as ● (*N=14*). Non-pregnant and baseline pregnant data analysed by Mann Whitney U test. Longitudinal data analysed by generalized linear mixed effects model with gamma log-link and pairwise multiple comparisons of estimated marginal means with sequential Bonferroni correction. P values are two tailed and significance is defined as * P<0.05, and ** P<0.01. A dashed line represents < 20 SFC/10^6.

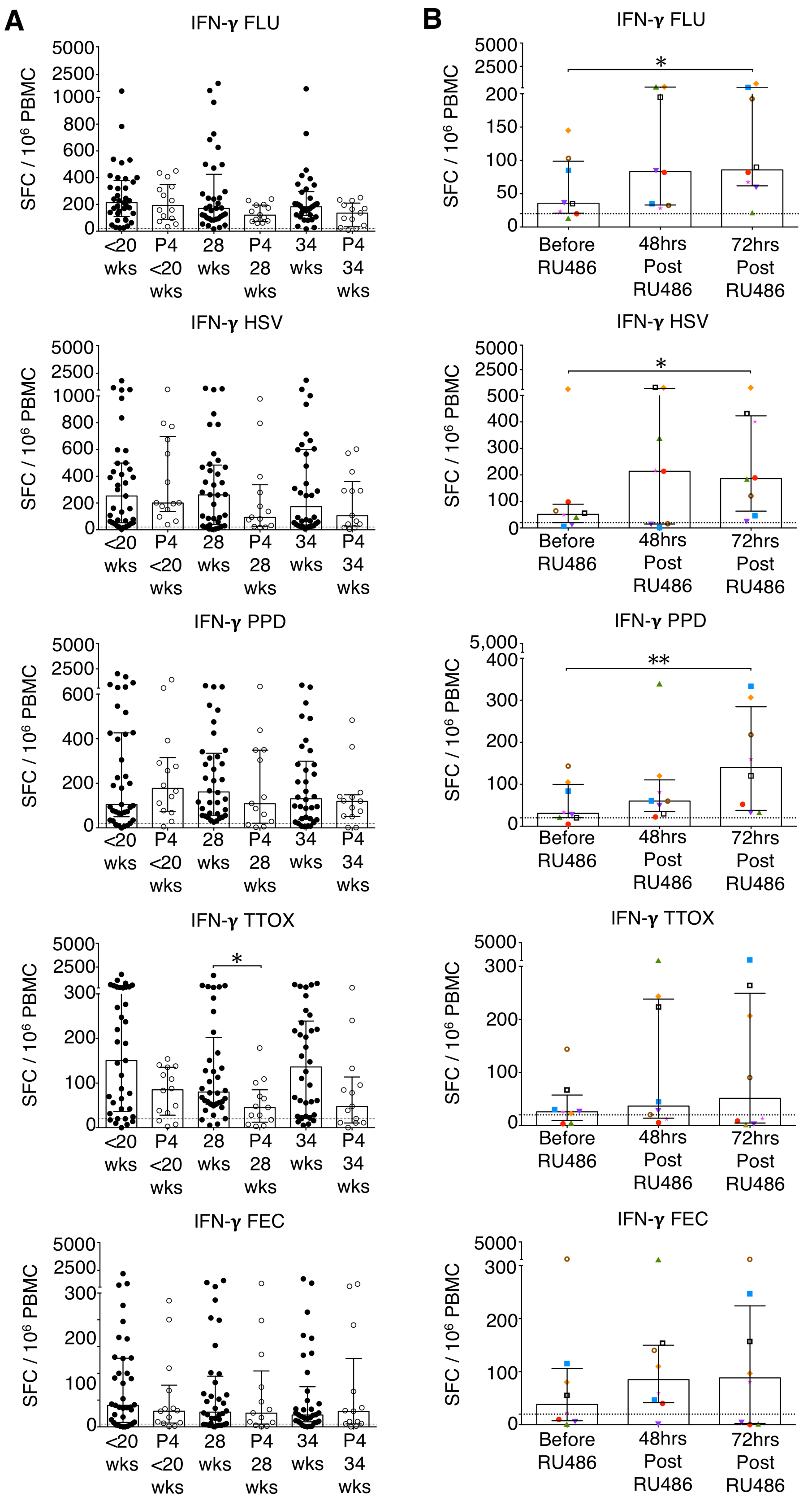

Figure S2. The influence of P4 and RU486 on IFN-γ ELISpot responses. (A) Gestation matched paired comparison of P4 treated versus untreated pregnant controls' IFN-γ ELISpot responses to influenza A and HSV whole lysates; PPD and TTOX antigens; and FEC peptide pool. Unpaired Mann Whitney U test. (B) Longitudinal analysis of RU486 treated pregnant patients' IFN-γ ELISpot responses to influenza A and HSV whole lysates; PPD and TTOX antigens; and FEC peptide pool. Freidman test with Dunn's post-hoc correction. Gestation at sampling is indicated: in pregnant controls (●) at <20^{+0} weeks (*N=42*), 28 weeks (*N=35*), 34 weeks (*N=33*), and P4 treated pregnancies (**O** *N=15*). For RU486 treated (*N=8*) symbols represent individual patients. P values are two tailed and significance is defined as * P<0.05, and ** P<0.01. A dashed line represents < 20 SFC/10^6.

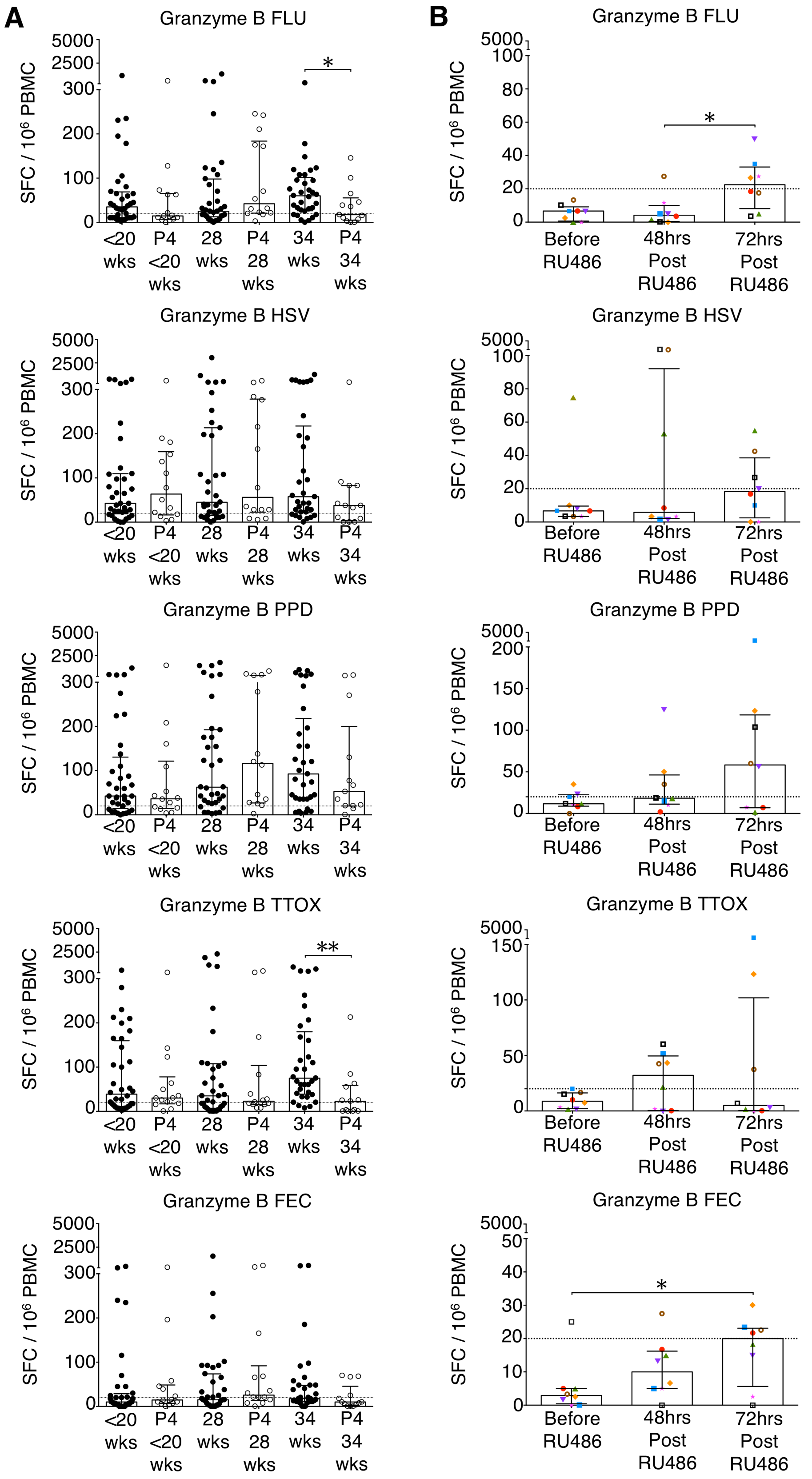

Figure S3. The influence of P4 and RU486 on Granzyme B ELISpot responses. (A) Gestation matched paired comparison of P4 treated versus untreated pregnant controls' Granzyme B ELISpot responses to influenza A and HSV whole lysates; PPD and TTOX antigens; and FEC peptide pool. Unpaired Mann Whitney U test. (B) Longitudinal analysis of RU486 treated pregnant patients' Granzyme B ELISpot responses to influenza A and HSV whole lysates; PPD and TTOX antigens; and FEC peptide pool. Freidman test with Dunn's post-hoc correction. Gestation at sampling is indicated: in pregnant controls (●) at $<20^{+0}$ weeks (*N=42*), 28 weeks (*N=35*), 34 weeks (*N=33*), and P4 treated pregnancies (○ *N=15*). For RU486 treated (*N=8*) symbols represent individual patients. P values are two tailed and significance is defined as * P<0.05, and ** P<0.01. A dashed line represents < 20 SFC/10^6.

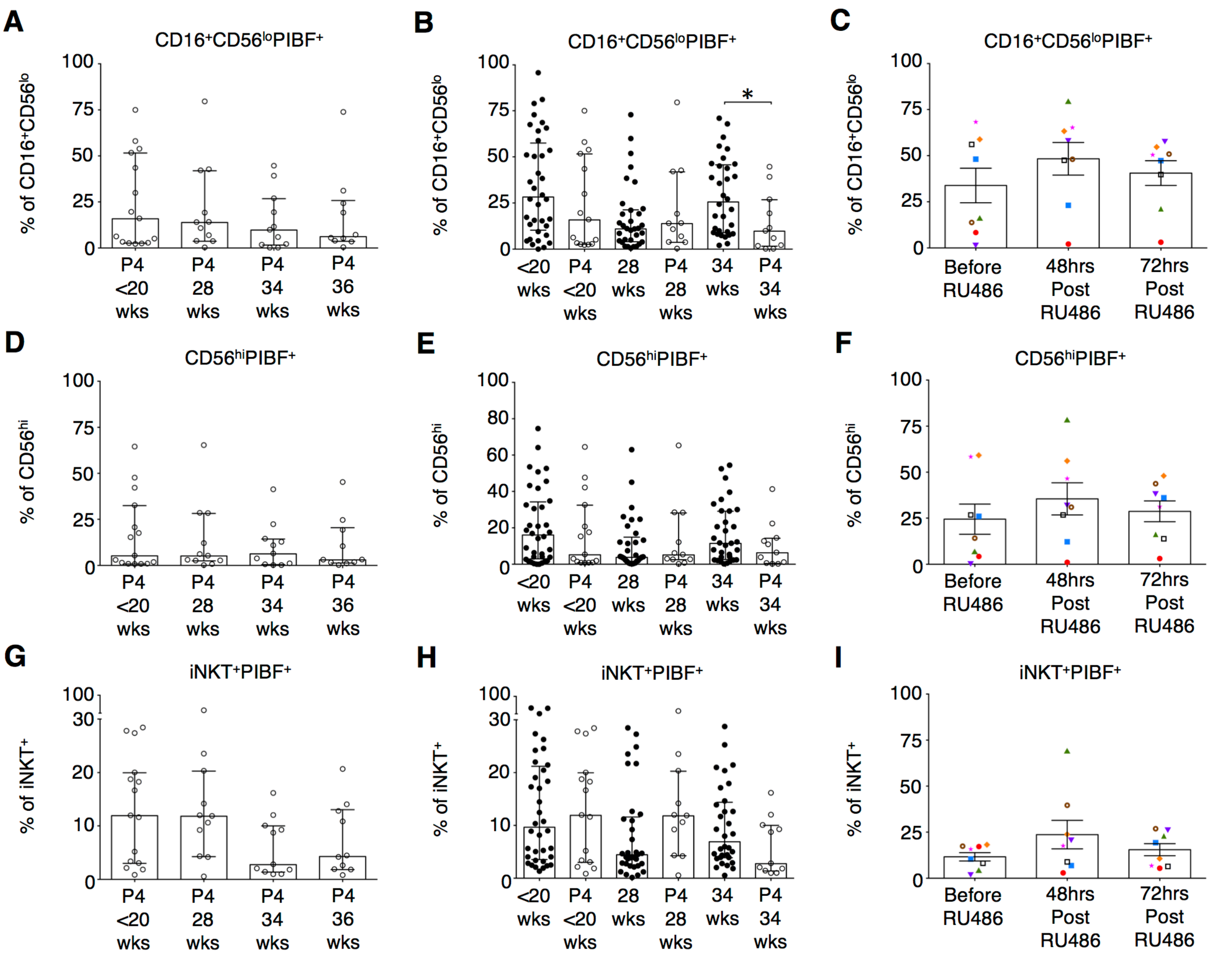

Figure S4. The effect of P4 and RU486 on PIBF expression on NK and iNKT cells. We show proportions of PIBF expressing $CD16^{+}CD56^{lo}$ NK cells in peripheral blood of P4 treated, measured (A) longitudinally, (B) compared with gestation matched controls, and (C) in RU486 treated measured longitudinally. In addition, proportions of PIBF expressing $CD56^{hi}$ NK cells in peripheral blood of P4 treated, measured (A) longitudinally, (B) compared with gestation matched controls, and (C) in RU486 treated measured longitudinally. Finally, Proportions of PIBF expressing $iNKT^{+}$ cells in peripheral blood of P4 treated, measured (A) longitudinally, (B) compared with gestation matched controls, and (C) in RU486 treated measured longitudinally. Gestation at sampling is indicated: in pregnant controls (●) at $<20^{+0}$ weeks (*N=42*), 28 weeks (*N=35*), 34 weeks (*N=33*), and P4 treated pregnancies (**○** *N=15*). For RU486 treated (*N=8*) symbols represent individual patients. P values are two tailed and significance is defined as * P<0.05.
